# Crowding in the emergency department in the absence of boarding – a transition regression model to predict departures and waiting time

**DOI:** 10.1186/s12874-019-0710-3

**Published:** 2019-03-29

**Authors:** Andreas Halgreen Eiset, Hans Kirkegaard, Mogens Erlandsen

**Affiliations:** 1 Department of Public Health, Aarhus University, Aarhus, Denmark; 2 Research Center for Emergency Medicine, Aarhus University Hospital, Aarhus, Denmark

**Keywords:** Emergency department, Crowding, Prediction model, Waiting time, Transition regression model

## Abstract

**Background:**

Crowding in the emergency department (ED) is associated with increased mortality, increased treatment cost, and reduced quality of care. Crowding arises when demand exceed resources in the ED and a first sign may be increasing waiting time. We aimed to quantify predictors for departure from the ED, and relate this to waiting time in the ED before departure.

**Methods:**

We utilised administrative data from the ED and calculated number of arrivals, departures, and the resulting queue in 30 min time steps for all of 2013 (*N* = 17,520). We build a transition model for each time step using the number of past departures and pre-specified risk factors (arrivals, weekday/weekend and shift) to predict the expected number of departures and from this the expected waiting time in the ED. The model was validated with data from the same ED collected March through August 2014.

**Results:**

We found that the number of arrivals had the greatest independent impact on departures with an odds ratio of 0.942 (95%CI: 0.937;0.948) corresponding to additional 7 min waiting time per new arrival in a 30 min time interval with an a priori time spend in the ED of two hours. The serial correlation of departures was present up to one and a half hour previous but had very little effect on the estimates of the risk factors. Boarding played a negligible role in the studied ED.

**Conclusions:**

We present a transition regression model with high predictive power to predict departures from the ED utilising only system level data. We use this to present estimates of expected waiting time and ultimately crowding in the ED. The model shows good internal validity though further studies are needed to determine generalisability to the performance in other settings.

**Electronic supplementary material:**

The online version of this article (10.1186/s12874-019-0710-3) contains supplementary material, which is available to authorized users.

## Background

Crowding in the emergency department (ED) can be defined as a situation where demand exceed the resource supply (i.e. beds, nurses, doctors etc.) and is associated with increased mortality, increased treatment cost, and reduced quality of care [1–7]. Asplin et al. has proposed a conceptual model of crowding that incorporates risk factors associated with crowding dividing patient flow in the ED into input (arrivals, high proportion of complex or critically ill patients and restricted access to general practice), throughput (internal processes, staffing, delay in test results), and output (departures, inpatient boarding) [7–9]. We have previously shown that crowding can be described with a general black box queuing model [10] and found that in particular the input to the ED drives the queue length (i.e. the number of patients in the ED) in a given 30 min time interval.

While several studies [11–15] have investigated patient-level factors (e.g. sex, age, ethnicity, and acuity level) associated with length of stay in the ED, analysing crowding in the ED using system-level data (e.g. day of week, work shift etc.) is a less explored approach [16, 17]. This method has the advantage that the investigated factors can be anticipated to a high degree and staffing can be planned accordingly.

## Methods

The aim of the present study was to model the probability of a departure from the ED dynamically as a function of the number of arrivals and the queue length given weekday or weekend and work shift (day, evening, or night) thus quantifying the effect of these previously identified risk factors for crowding in the ED [10]. We will use the model to estimate the expected waiting time in the ED – from arrival to departure – under different scenarios. We adhere to the Strengthening the Reporting of Observational Studies in Epidemiology statement (https://www.strobe-statement.org).

### Study site

We obtained data from the ED unit at Aarhus University Hospital, Denmark. The ED attends to approximately 40,000 patients per year from an uptake area of 330,000 inhabitants and receives all acute orthopaedic, trauma, and unstable medical patients from this area. Patients with other surgical and medical needs are also attended in the studied ED though not exclusively in this unit. Patient groups that are not received in the ED include medical paediatric and psychiatric patients as well as patients with cardiac arrest and myocardial infarction (these were transferred to specialised departments). When admitted to the ED unit patients are treated and discharged or admitted to the hospital. See Additional file 1 for details of the studied ED and the study population.

### Study design and population

We used an open cohort design including patients from 1. January 2013 to 31. December 2013. From the Electronic Health Records (EHR) we obtained data on arrivals to and departures from the ED including time stamps. The total number of arrivals was 41,693.

We created an aggregate data set with census of arrivals A(t) and departures D(t) in 30 min intervals (*N* = 24 * 2 * 365 = 17,520). The queue length at the beginning of a new interval (t + 1) was calculated from the queue length, number of arrivals and departures in the previous interval (t):

$$ \mathrm{Q}\left(\mathrm{t}+1\right)=\mathrm{Q}\left(\mathrm{t}\right)+\mathrm{A}\left(\mathrm{t}\right)-\mathrm{D}\left(\mathrm{t}\right), $$

with the initial queue length Q(0) = 0.

To mirror the clinical setting we defined a day as beginning with the day shift at 7 a.m. with each work shift lasting eight hours. To reduce the effect of the arbitrary choice of the initial queue length (Q(0) = 0) the first three shifts of the study period were excluded in the statistical analysis.

### A statistical model for the number of departures

We expected serial or lagged correlation of the departures (correlation of two observations of departure as a function of the time between them, also known as “autocorrelation”) and thus utilised a transition model for each time step (present departures) with conditional analyses on past departures (as predictor variables) together with the pre-specified predictor variables (risk factors) to predict the expected number of present departures [18]. The transition model is formulated as a binomial regression model for each time step t, where the number of trials is equal to the maximum number of possible departures (n(t) = Q(t) + A(t)) and the number of successes corresponds to the actual number of departures D(t), i.e. D(t) ~ Bin(n(t), p(t | t-1)), where p(t *|* t-1) is the probability of a departure in time step t dependent on past values.

In binomial regression models it is standard to express the probability p for given values of the predictors through the logit function (as in logistic regression):

$$ \ln \left(\mathrm{p}/\left[1\hbox{-} \mathrm{p}\right]\right)=\alpha +\beta (1)\times \mathrm{x}(1)+\beta (2)\times \mathrm{x}(2)+\dots $$

In our setting we used this expression for each time step with predictors that include both the number of past departures to handle the time dependent transition step and the pre-specified predictor variables

$$ {\displaystyle \begin{array}{l}\ln \left(\mathrm{p}\left(\mathrm{t}+1\ |\ \mathrm{t}\ \right)/\left(1\hbox{-} \mathrm{p}\left(\mathrm{t}+1\ |\ \mathrm{t}\right)\right)\right)=\alpha \\ {}\kern4.199998em +\delta (11)\times \mathrm{D}\left(\mathrm{t}\right)+\delta (12)\times \mathrm{D}\left(\mathrm{t}\hbox{-} 1\right)+\dots \kern0.72em \left(\mathrm{p}\mathrm{ast}\ \mathrm{departures}\right)\\ {}\kern4.199998em +\beta (1)\times \mathrm{x}(1)+\beta (2)\times \mathrm{x}(2)+\beta (3)\times \mathrm{x}(3)+\dots \left(\mathrm{p}\mathrm{re}\hbox{-} \mathrm{specified}\kern0.17em \mathrm{p}\mathrm{re}\mathrm{dictors}\right)\end{array}} $$

Due to the logit model each regression parameter can be expressed as an odds ratio (OR) by exp(β(i)) = OR(i).

The pre-specified predictor variables were new arrivals, weekday/weekend and shift (day, evening, or night) since these have been shown to be important risk factors for crowding [10]. We also included queue length in the previous time interval as well as the change in queue length to the current time interval. Our previous study indicated interaction between weekday/weekend and work shift why this was included in the model as well. We tested the overall interaction by chi^2 test (ANOVA method).

### Relating the model to waiting time

We will use “waiting time” in the meaning of “waiting to leave the ED”, synonymously with the term “length-of-stay” (ED LOS), counting from the time of arrival until the time of departure from the ED disregarding the fact that much imperative work - such as assessment, treatment etc. - is being done during this time. Thus, here “waiting time” is a matter of queueing terminology and should not be taken literally.

Assuming that the queue length is in steady state the number of departures will follow a geometric distribution and hence the expected time spend from arriving to the ED in a given 30 min time interval until leaving the ED (immediate waiting time) can be estimated in hours as

$$ \mathrm{WT}=0.5\kern0.28em \times \left(1-\mathrm{p}\right)/\mathrm{p}, $$

where p is the probability of a departure. The ratio between waiting time in two scenarios with departure probabilities p(1) and p(0) is

$$ {\displaystyle \begin{array}{l}\mathrm{WT}(0)/\kern0.28em \mathrm{WT}(1)=\\ {}\mathrm{OR}\left({}^{"}{0}^{"}\mathrm{vs}.{}^{"}{1}^{"}\right),\end{array}} $$

that is, the inverse OR between the departure probabilities can be interpreted as the factor by which the expected waiting time changes.

### Diagnostics

From the binomial model we calculated for each time step the expected number of departures (fitted) as E(t + 1) = E(D(t + 1)) = n(t + 1) * p(t + 1 | t), where p(t + 1 | t) is considered as the one-step-ahead prediction for the probability of departure and is estimated through the logit-expression given above. We plotted the standardised residuals against the fitted values to check for the assumptions of linearity, homoscedasticity, and independent errors (Fig. 2). To confirm normality of the residuals we plotted the standardised residual quantiles against the theoretical quantiles (“Normal Q-Q plot”, Fig. 2). We evaluated the goodness-of-fit by the null and residual deviance.

### Model validation

We used a new dataset from the same ED but collected during 6 month of 2014 (March 1st to August 31st) to evaluate how well our final model captures the observed data. We evaluated the model fit the same way as for the model presented above but using parameter estimates from the original data (Additional file 2).

Data management, analysis and plots were done in R, version 3.3.2 (R Foundation for Statistical Computing, Vienna, Austria). The code is available together with a constructed example of the dataset in the GitHub repository https://github.com/eiset/Crowding_code.git

## Results

In the study period, approximately 3% of the patients stayed less than 30 min in the ED and 5% stayed longer than 5 h. The ED had 19 beds and was staffed with 4 to 8 nurses depending on time of day and week. A detailed description of the study population and site can be found in [10].

The serial correlation on previous departures was present up to one and a half hour previous (OR = 1.008 to 1.012).

New arrival (OR = 0.942) and change in queue length (OR = 0.978) had the greatest impact on lowering the odds of a departure. In a “standard scenario” of weekday day shift, with an empty queue for at least the past one and a half hour the waiting time for one patient arriving to the ED is given by the transition model as

$$ {\displaystyle \begin{array}{l}\mathrm{WT}(0)=\\ {}0.5\kern0.28em \times \kern0.28em 1/\exp \left(\alpha +{\beta}_{\mathrm{Arrival}}\right)=\\ {}0.5\kern0.28em \times \kern0.28em 1/\exp \left(-1.397+-0.059\right)=\\ {}2.15\kern0.28em \mathrm{hours}.\end{array}} $$

See Table 1 for all OR estimates including confidence intervals and the corresponding impact on waiting time.

**Table 1:** Results of the transition regression model

	OR (95%CI)	*P*-value	Relative change in waiting time^a^
New arrival, A(t)	0.942 (0.937;0.948)	< 0.0001	106%
Change in queue length, Q(t) - Q(t-1)	0.978 (0.973;0.984)	< 0.0001	102%
Queue length in past time interval, Q(t)	0.991 (0.989;0.993)	< 0.0001	101%
Departure in time interval half an hour previous, D(t)	1.008 (0.999;1.016)	0.0596	99%
Departure in time interval one hour previous, D(t-1)	1.012 (1.007;1.018)	< 0.0001	99%
Departure in time interval one and a half hour previous, D(t-2)	1.009 (1.004;1.015)	0.0004	99%
Shift			
Day	1		
Evening	1.103 (1.070;1.137)	< 0.0001	91%
Night	1.145 (1.092;1.202)	< 0.0001	87%
Weekday/weekend			
Weekday	1		
Weekend	1.002 (0.960;1.046)	0.9234	100%
Shift x weekday/weekend			
Day x weekday	1		
Evening x weekend	1.152 (1.092;1.216)	< 0.0001	87%
Night x weekend	0.943 (0.879;1.012)	0.1009	106%

^a^The change in expected waiting time corresponding to the OR. Abbreviations: *OR* odds ratio, *95%CI* 95% confidence interval

Weekday/weekend did not have an isolated effect but did show interaction with work shift (overall test for interaction *p* <  0.0001) with a higher probability for a departure in weekends evening and night shift as compared to weekday day shift (see Table 2).

**Table 2:** Interaction of work shift and weekday/weekend, odds ratio

	Weekday^a^	Weekend^a^
Day shift	1	1.002 (100%)
Evening shift	1.103 (91%)	1.273 (79%)
Night Shift	1.145 (87%)	1.082 (92%)

Overall test for interaction *p* < 0.0001. ^a^Numbers in parentheses gives the expected change in waiting time corresponding to the odds ratio

**Fig. 1:**
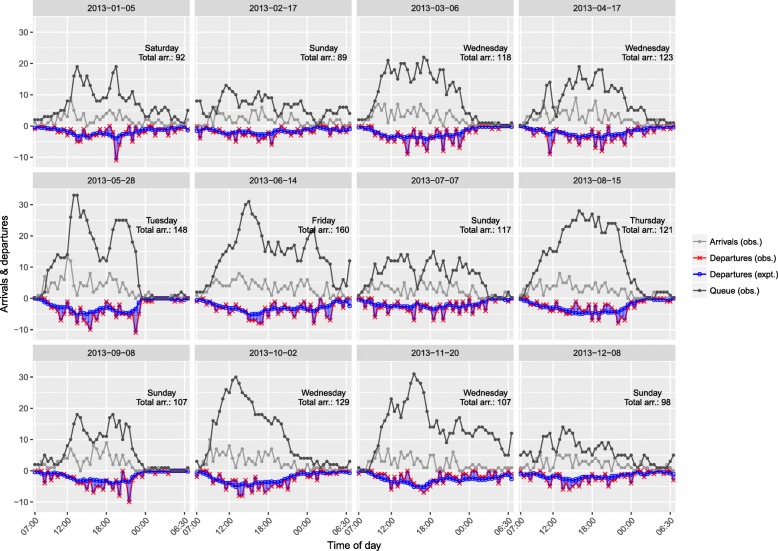
Arrivals, departures (observed and expected), and queue length. The 12 days have been randomly chosen (one for each month). Departures are plotted as negative for visualisation. Abbreviations: obs., observations; expt., expected; arr., arrivals

From Fig. 1 of the one-step-ahead expected and the observed number of departures (together with number of arrivals and the resulting queue length) we find that the model predicts departures fairly well with a close relation to the observed development over time. The random variation in the observed numbers around the time curve of the expected numbers show no clear evidence against the model, e.g. systematic over- or underfitting (see also Additional file 3 for more figures of randomly chosen days).

**Fig. 2:**
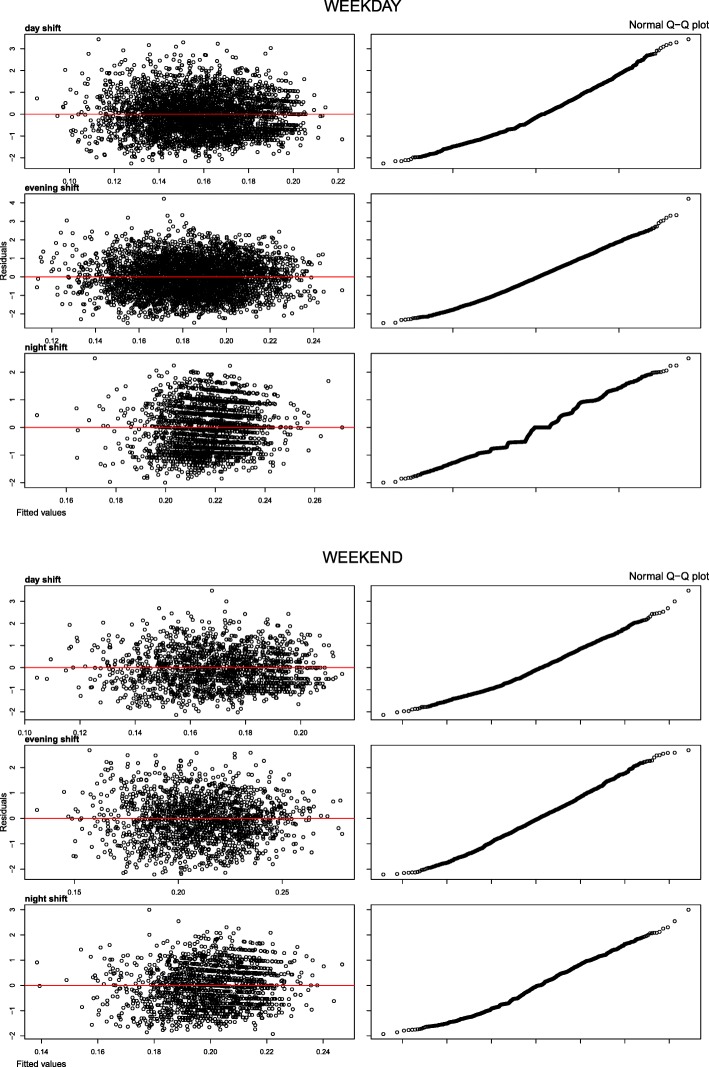
Diagnostics: Residuals against fitted values and QQ-plots of residual and theoretical quantiles. Stratified on shift and weekday/weekend. Residuals against fitted values plot: Linearity indicated by a symmetric distribution around zero with no trend. Homoscedasticity indicated by no trend in the vertical scatter. Independent errors indicated by lack of any pattern. QQ-plot: To check for normality

All diagnostic plots suggested that the binomial model adhered to the assumptions underlying the model (Fig. 2).

## Discussion

We present a transition model quantifying predictors for departures from the ED taking into account the serial correlation of departures. The number of arrivals has the greatest effect on the departure probability and hence the waiting time. With only 5% of patients spending more than 5 h in the ED boarding was not a concern in the studied ED.

The expected waiting time rises with 6% – corresponding to around 7 min with an average a priori waiting time of two hours – per additional arrival in a 30 min time interval. Under this scenario, everything else being equal, the expected waiting time with three new arrivals will rise from 2 h to 2 h and 22 min in the duration of 30 min. Figure 1 shows that on 28. May at 12:30 the queue length rose to 26 patients. In the previous time intervals the queue length was 14 and the average waiting time was estimated to 4 h and 18 min data not shown). Everything else being equal, the previous queue length of 14 results in approximately 36 min additional waiting time in the time interval beginning at 12:30. To add to this, the queue length’s increase of 12 patients from the beginning of the previous time interval to the beginning of the interval at 12:30 gives a further increase of approximately 60 min waiting time. Additional file 4 shows a table of the development in expected waiting time for a day with few (80) arrivals and a day with many (147) arrivals. We also include a box-plot for comparison of the average waiting time in the two scenarios (Additional file 5).

The number of new arrivals may be associated with a broad range of predictable e.g. large festivals and recognised epidemics) and unpredictable (e.g. accidents and unrecognised epidemics) events. This number will, to a high degree, be random and hence hard to predict and to base any action upon. Contrary, a rise in the queue length as compared to the past 30 min time interval is easily monitored and is associated with a decreased probability of a departure as well. It indicates a vicious cycle beginning (growing queue length - > fall in departures/longer waiting time - > growing queue length - > …) and may very well be an early sign of crowding in the ED which can be countered. The Plan-Do-Study-Act tool could be utilised in implementing such intervention [19].

The pre-determined predictors’ coefficients were nearly unchanged when we did not include the serial (departure) correlation in the model. This indicates that it is to a lesser degree the throughput processes that affects whether or not a patient is likely to spend a long time in the ED: The “internal system” (throughput processes) remains the same through 30 min intervals. This is in accordance with our previous results and other studies [10, 11] though not unambiguously reported [14, 20].

Contrary to the conclusion of Bashkin et al. [16] we found no indication that departures or queue length is associated with shift changes (Fig. 1). This could be due to a more appropriate structuring of the hand-over between shifts in the studied ED or that Bashkin et al. have exclusively looked at throughput processes to explain departures and crowding and thus not included other confounders in their analysis. Wiler et al. [17] find an independent effect of weekend (compared to weekday), which we could not reproduce. We did however find an effect of the interaction of shift and weekend. It may be this interaction that was not investigated in the Wiler study or it may be that the organisation of the EDs under study or their respective uptake population is different in a way that produce the divergent result.

The chosen binomial model assumes that the probability of departure is homogeneous across patients in a given time interval, but it seems likely, that heterogeneity in e.g. triage scores (red patients) can cause heterogeneity in the departure probabilities. Due to the number of missing values on the triage score this predictor could not be included in the model.

All diagnostics as well as Fig. 1 (and Additional file 3) indicate that the model describes the data well. Our validation of the model showed an equally good fit indicating a high internal validity [21–23]. There may however still be a question of the external validity to other settings: EDs that are substantially different organised, different population characteristics in the uptake area, etc. Further validation studies are needed to determine this.

The EHR contains prospectively collected data to be used in a clinical setting. This makes EHR a secondary data source when used in research: We (the researchers) had no control over the data collection process, which might make EHR data questionable for research [24]. The time stamps for arrival and departure is considered to be very reliable in the studied ED and any errors are likely to be random. If systematic error exists - e.g. if at times of increasing strain on the ED staff the time stamps of departures were registered with delay (i.e. differential misclassification [23]) - this would lead to bias in the reported OR for departures (in the example given it would lower the OR related to queue length). Other possible predictors on a system-level to include in the model is season, weather condition, epidemics (e.g. influenza), large events in the area (e.g. music festivals), staffing of personnel other than nurses (e.g. doctors), and organisational and psychological interactions that may particularly play a role in times of increased strain on the staff such as in situations with crowding [25]. We have previously found that season plays a minor role in predicting crowding [10] and did not have data on the other variables to investigate their effect. Predictors for crowding on patient-level have been thoroughly examined and include age, sex, and acuity level of patients present in the ED [11–14, 26] but could not be included in the presented model (see above).

## Conclusions

We present a regression model to predict departures from the ED in the absence of boarding. We use this to present estimates of expected waiting time and ultimately crowding in the ED. Our model follows the recommendations by McCarthy et al. [27] for measuring crowding: It is highly generic – requiring data on only arrivals and departures – and dynamic with measurements in 30 min time steps.

## Additional files

Additional file 1: Table of characteristics of the emergency department and the patients. Friday and Saturday nights were considered part of the weekend. *The ED unit has two additional beds reserved for trauma call patients. Adapted from Eiset et al. (TIF 1490 kb)

Additional file 2: Figure of diagnostic plot for the validation data (six month of 2014). See main text, Figs. 1 and 2, and Additional file 3 for comparison to 2013 data. See Additional file 3 for plots of arrivals, departures and queue length. (PDF 1495 kb)

Additional file 3: Figure of arrivals, departures (observed and expected), and queue length in the study period. The 12 days have been randomly chosen (one for each month in 2013) eight times. Departures are plotted as negative for visualisation. Abbreviations: obs., observations; expt., expected; arr., arrivals. (PDF 1111 kb)

Additional file 4: Table of the expected waiting time in a scenario with few and many arrivals. Two examples of the number of arrivals and departures, the resulting queue length and probability of departure, and the waiting time estimate based on this data. The examples are chosen to show the contrast of a day with few arrivals and a day with many arrivals (the graphs of predicted arrivals etc. can be seen in Additional file 3). It exemplifies how arrivals can drive the waiting time e.g. in the time interval beginning at 15:00: The queue length were the same and twice as many (2 and 4, respectively) left the ED on the 5th September. But while there were four patients that arrived on the 5th eight arrived on the 20th. This resulted in a waiting time estimate of 2 h and 39 min and 1 h and 59 min, respectively. It is also clear that arrivals are not the only factor that influence waiting time. The change in queue length is seen to be important exemplified in the time intervals beginning at 7:30 and 8:00 both on the 5th September. Here nothing but the queue length changed (rises from 1 to 3 patients, respectively) and the waiting time increasing with 5 min. See Additional file 5 for a boxplot of the waiting time. *Waiting time estimated in hours as 0.5 * (1 – p) / p, where p is the probability of a departure. (PDF 41 kb)

Additional file 5: Boxplot, illustrating the median and inter quartile range (IQR) of the waiting time on the 20th October and 5th September 2013. The median estimated waiting time was 1 h and 48 min (IQR = 27 min) on the 20th October and 2 h and 13 min (IQR = 40 min) on the 5th September. The notch indicates the estimated 95% confidence interval for the median. The individual observations are jittered. See Additional file 4 for a table of arrivals, departures and expected waiting time for each 30 min time interval. (TIF 425 kb)

## Abbreviations

95%CI: 95% confidence interval
A: Arrivals
D: Departures
E: Expected
ED: Emergency department
EHR: Electronic health record
LOS: Length-of-stay
OR: Odds ratio
t: Time interval
WT: Waiting time
