# Supplementary figures and images for: Crowding in the emergency department in the absence of boarding – a transition regression model to predict departures and waiting time
Source: BMC Med Res Methodol. 2019 Mar 29;19:68. doi: 10.1186/s12874-019-0710-3 (PMC6440135; doi:10.1186/s12874-019-0710-3)

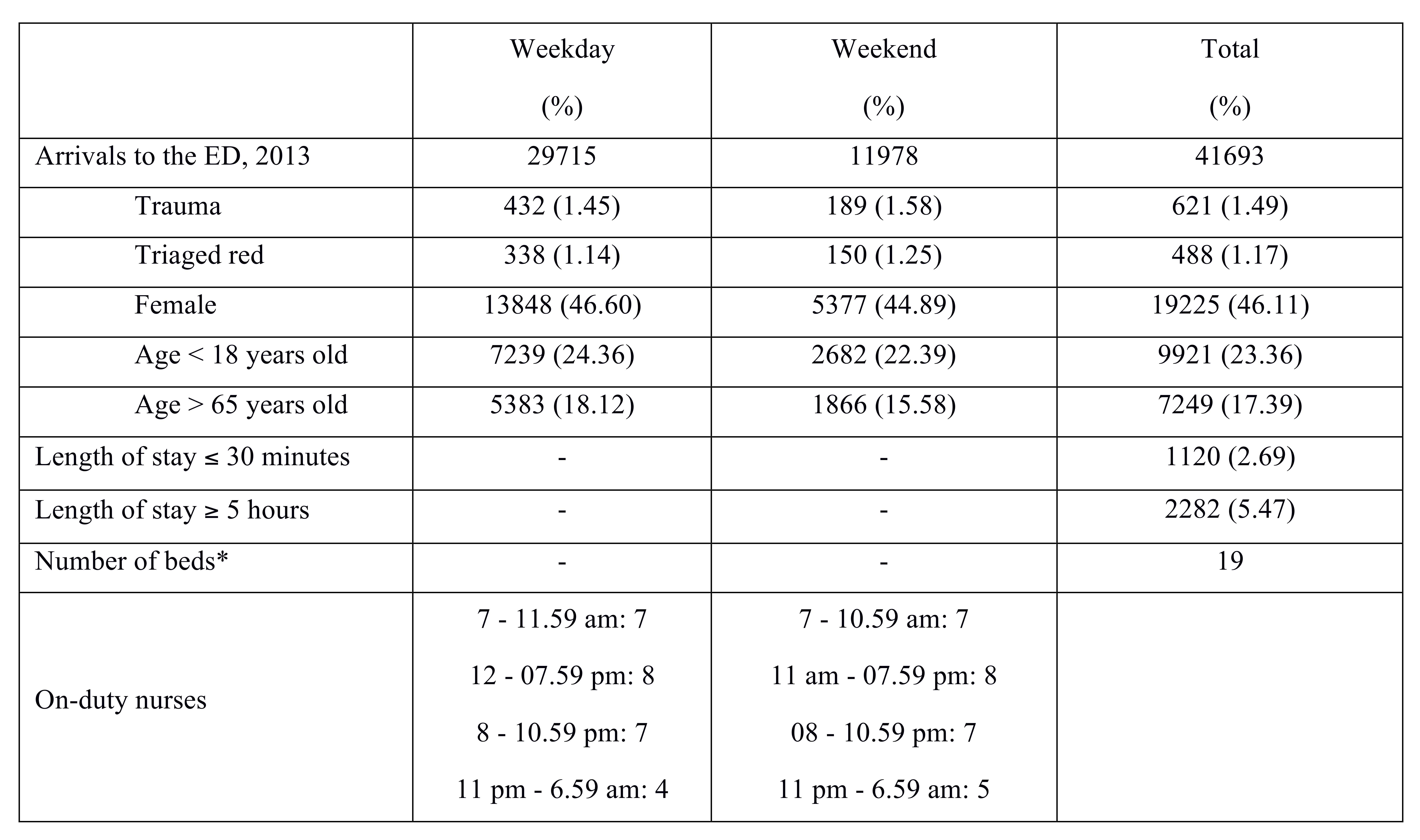

Supplement: Supplementary file 1 — Table of characteristics of the emergency department and the patients. Friday and Saturday nights were considered part of the weekend. *The ED unit has two additional beds reserved for trauma call patients. Adapted from Eiset et al. (TIF 1490 kb) [file 12874_2019_710_MOESM1_ESM.tif]

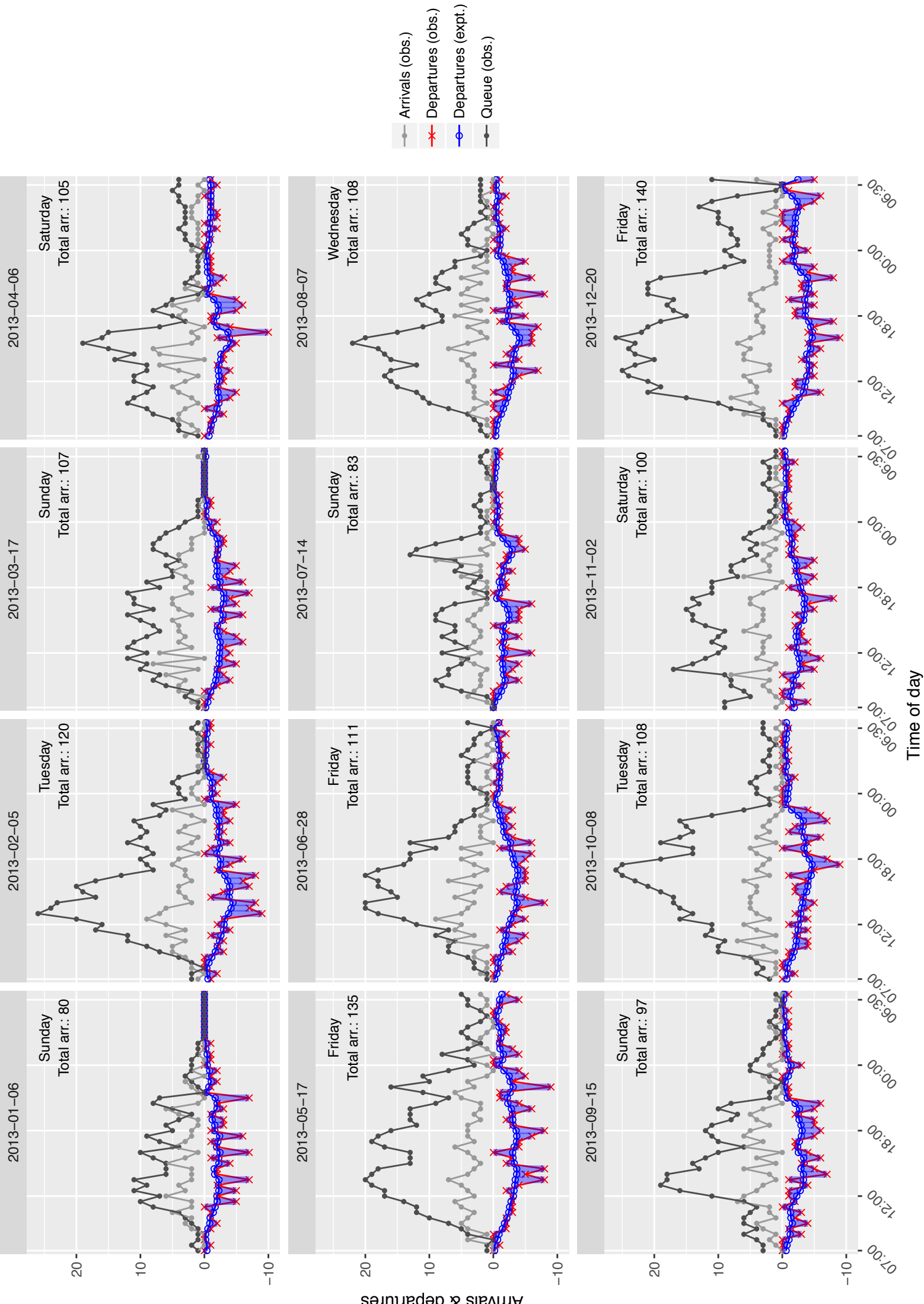

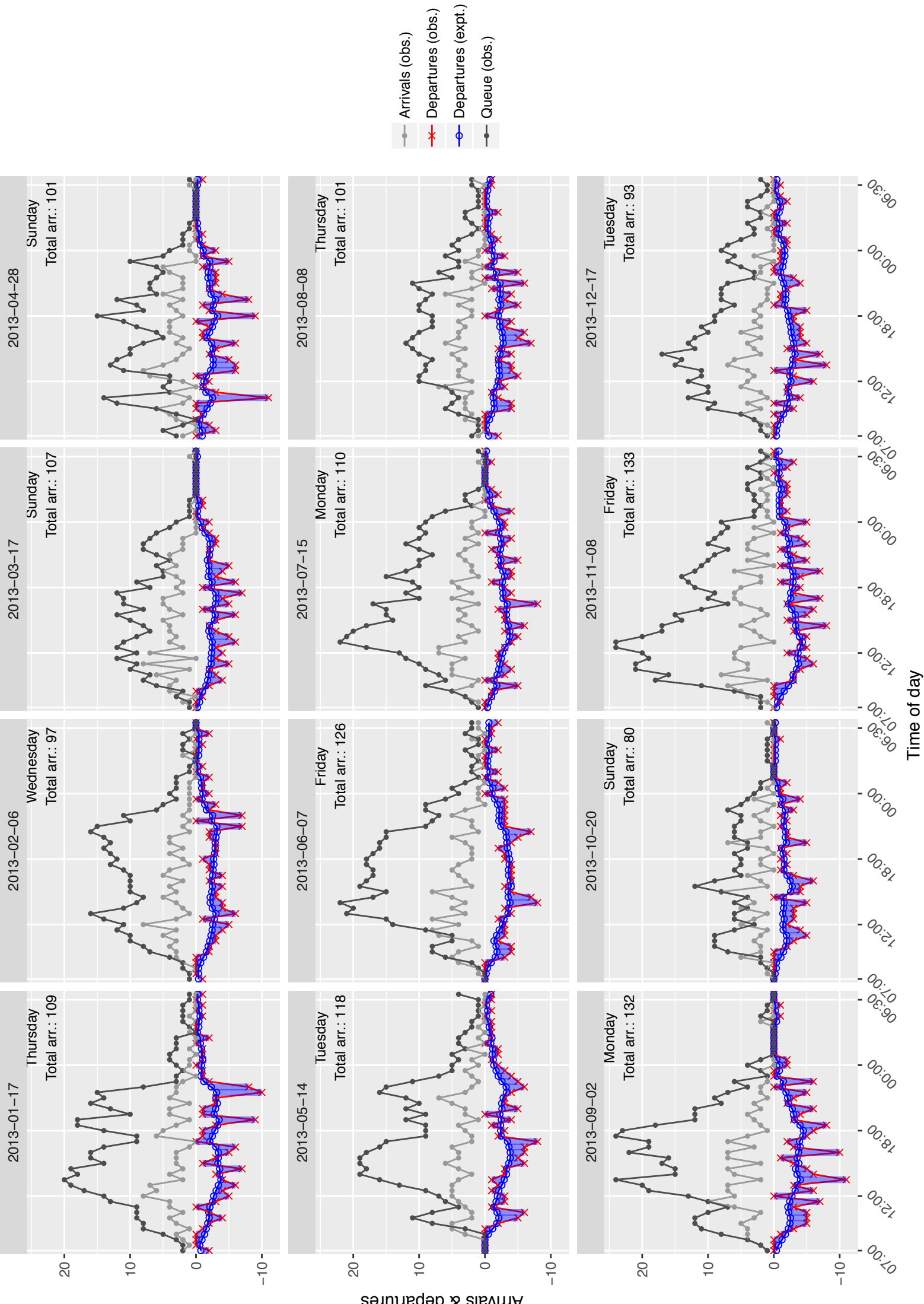

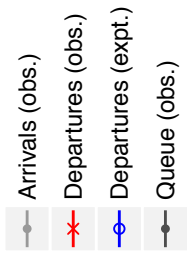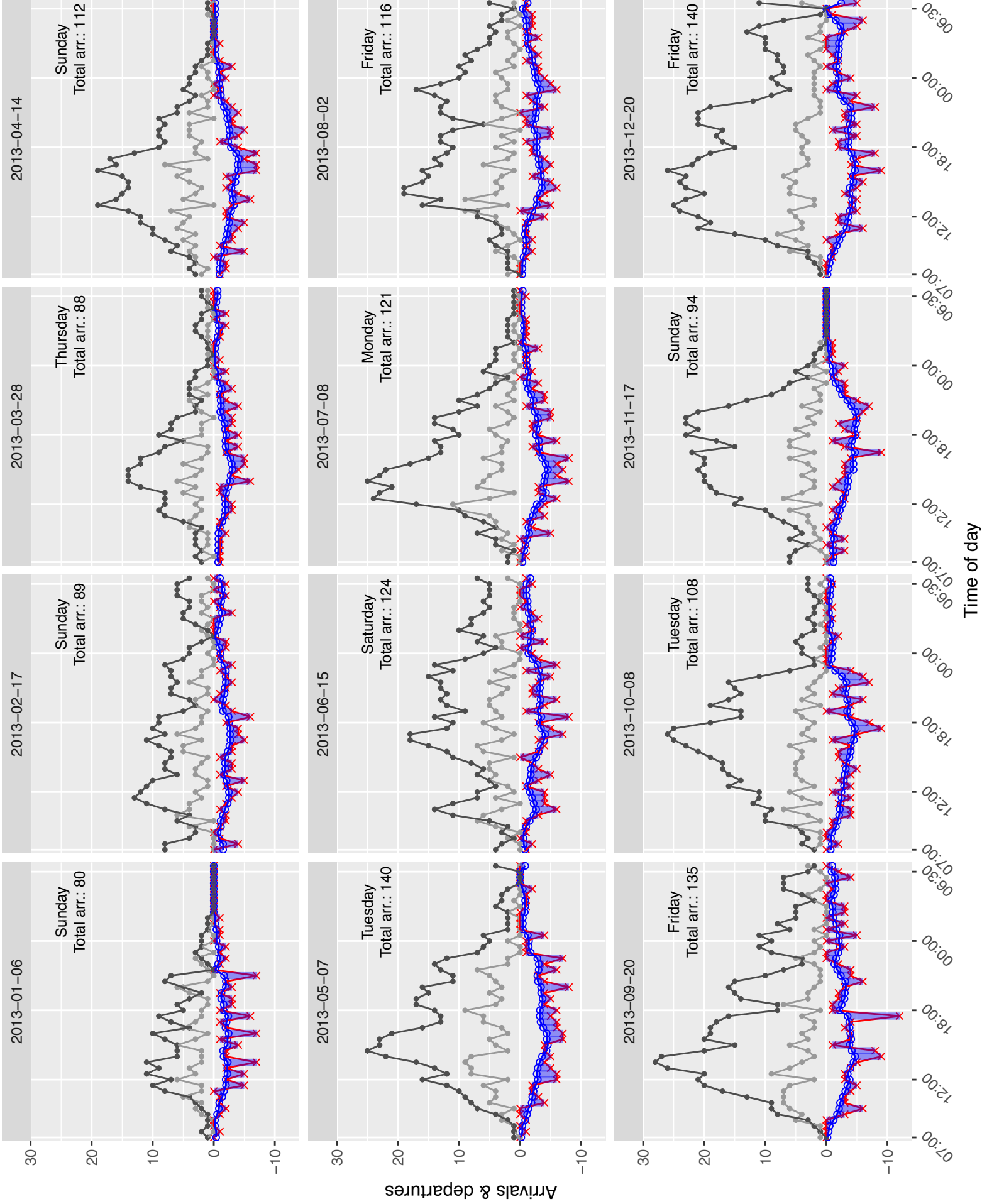

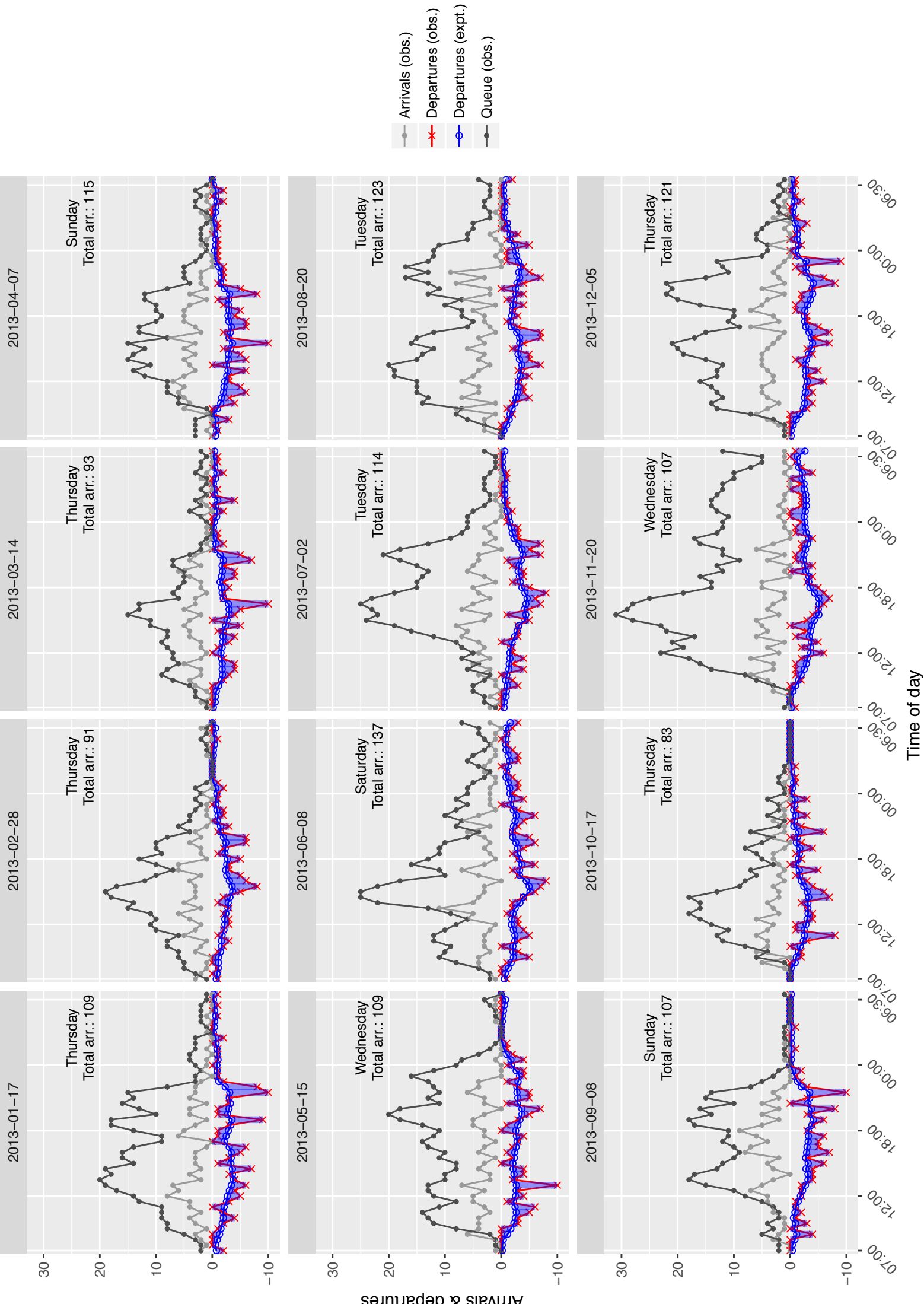

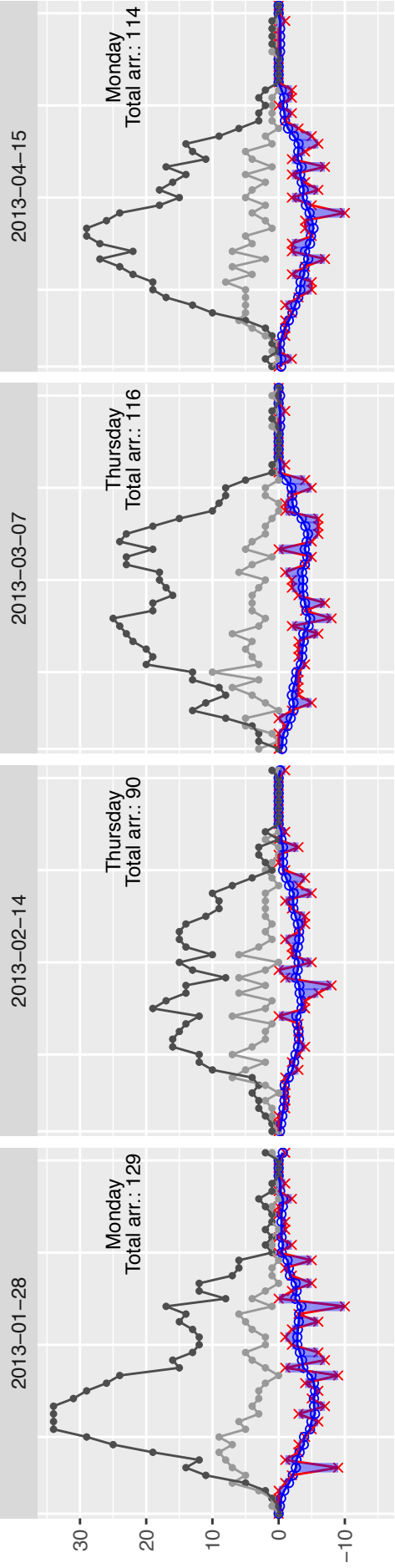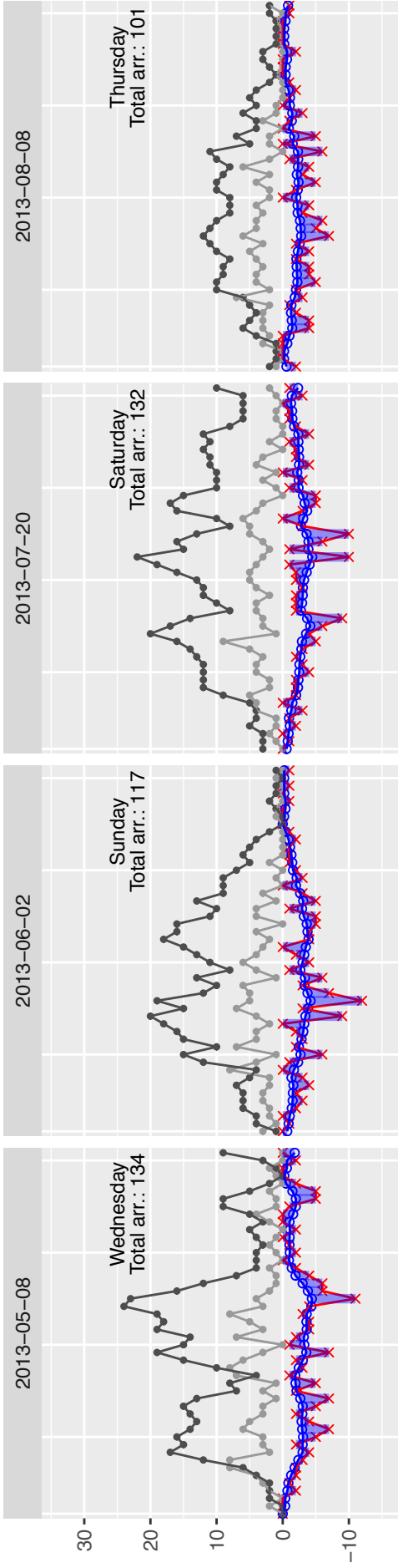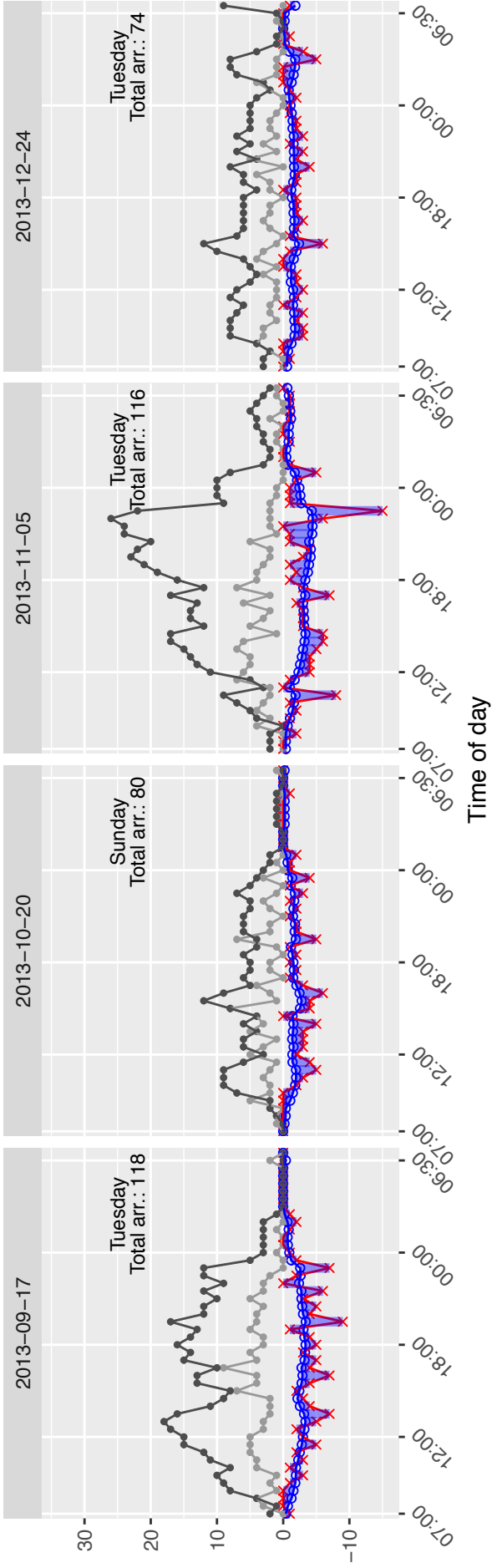

Arrivals (obs.)

Departures (obs.)

Departures (expt.)

Queue (obs.)

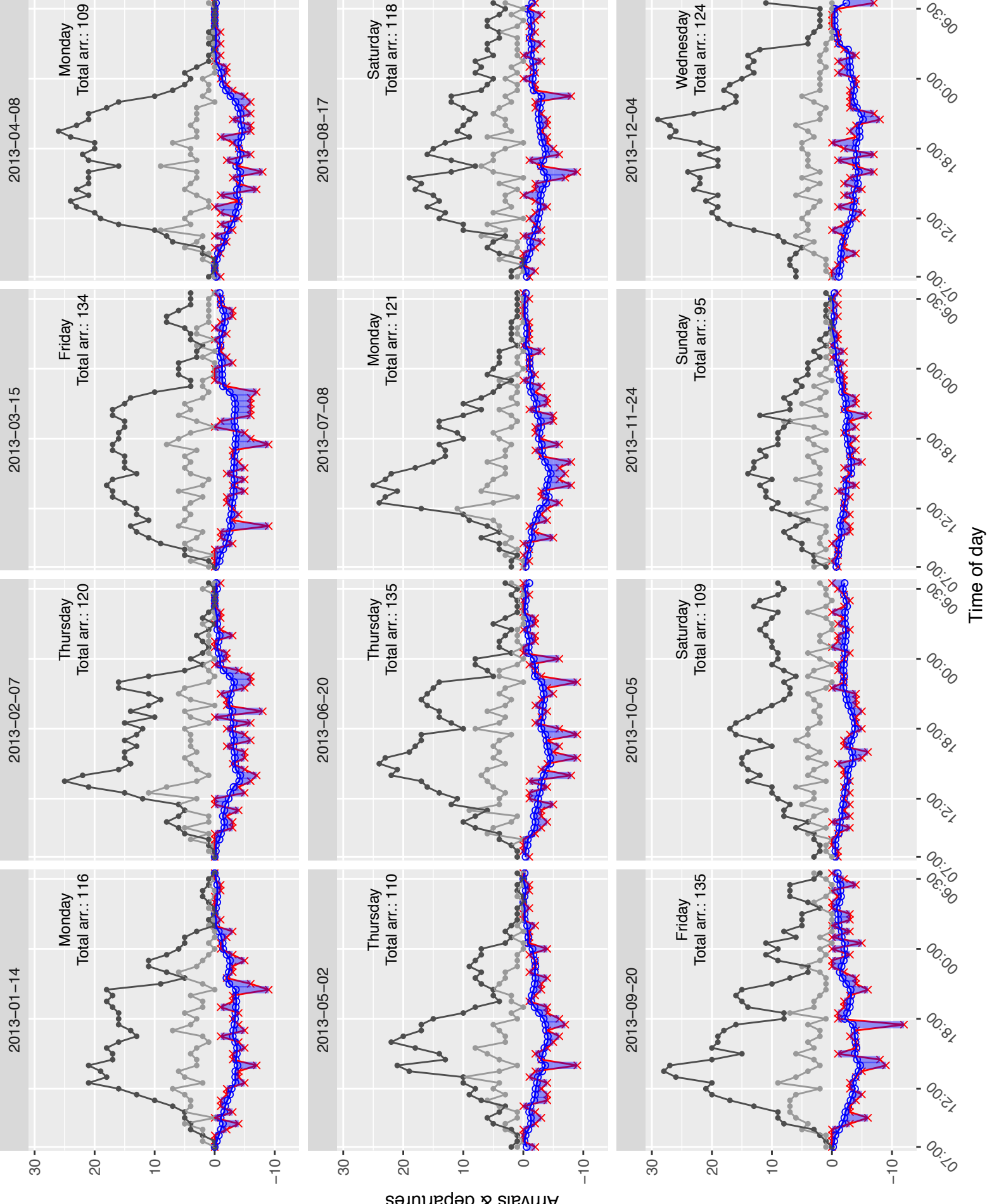

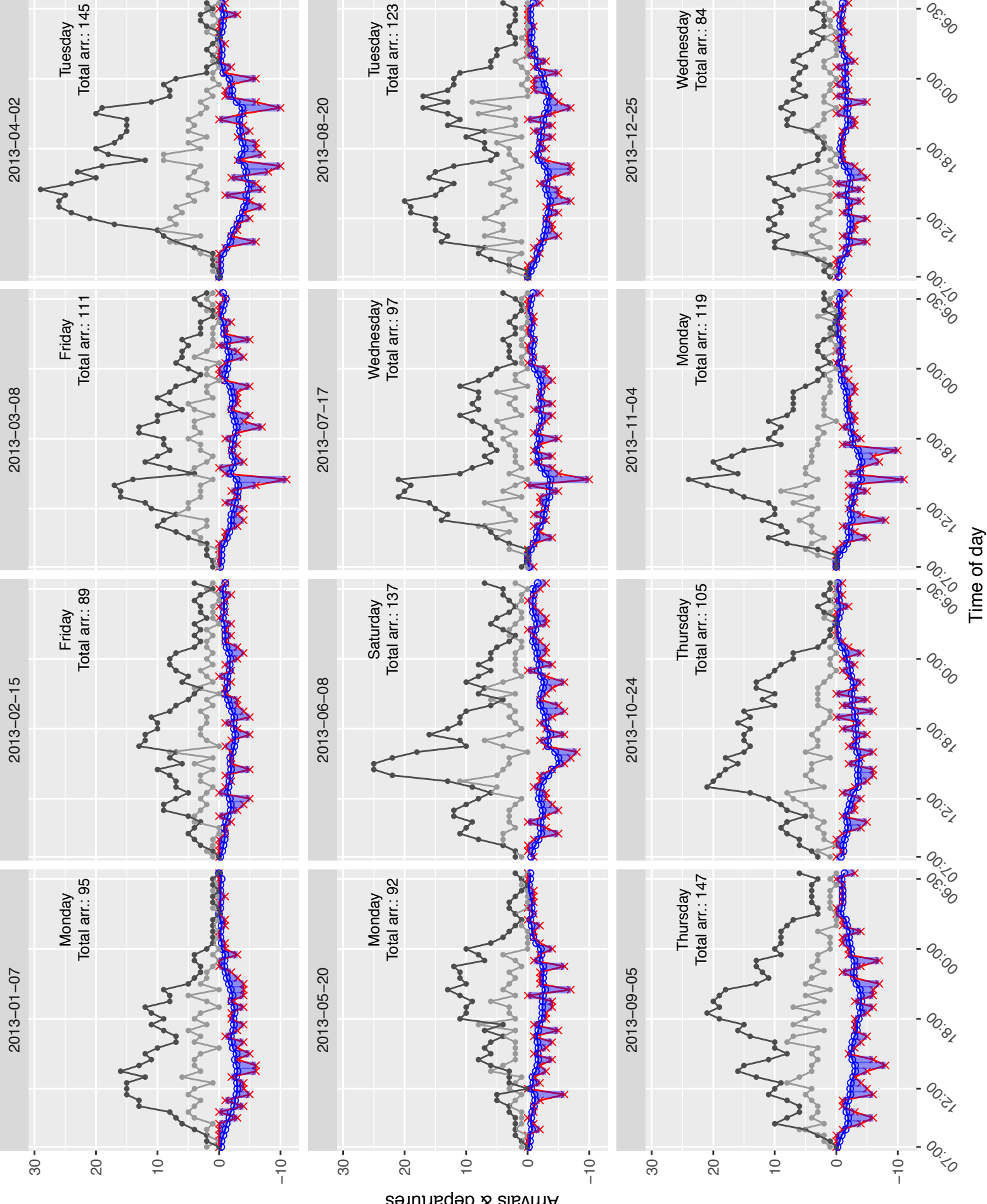

Supplement: Supplementary file 3 — Figure of arrivals, departures (observed and expected), and queue length in the study period. The 12 days have been randomly chosen (one for each month in 2013) eight times. Departures are plotted as negative for visualisation. Abbreviations: obs., observations; expt., expected; arr., arrivals. (PDF 1111 kb) [file 12874_2019_710_MOESM3_ESM.pdf]

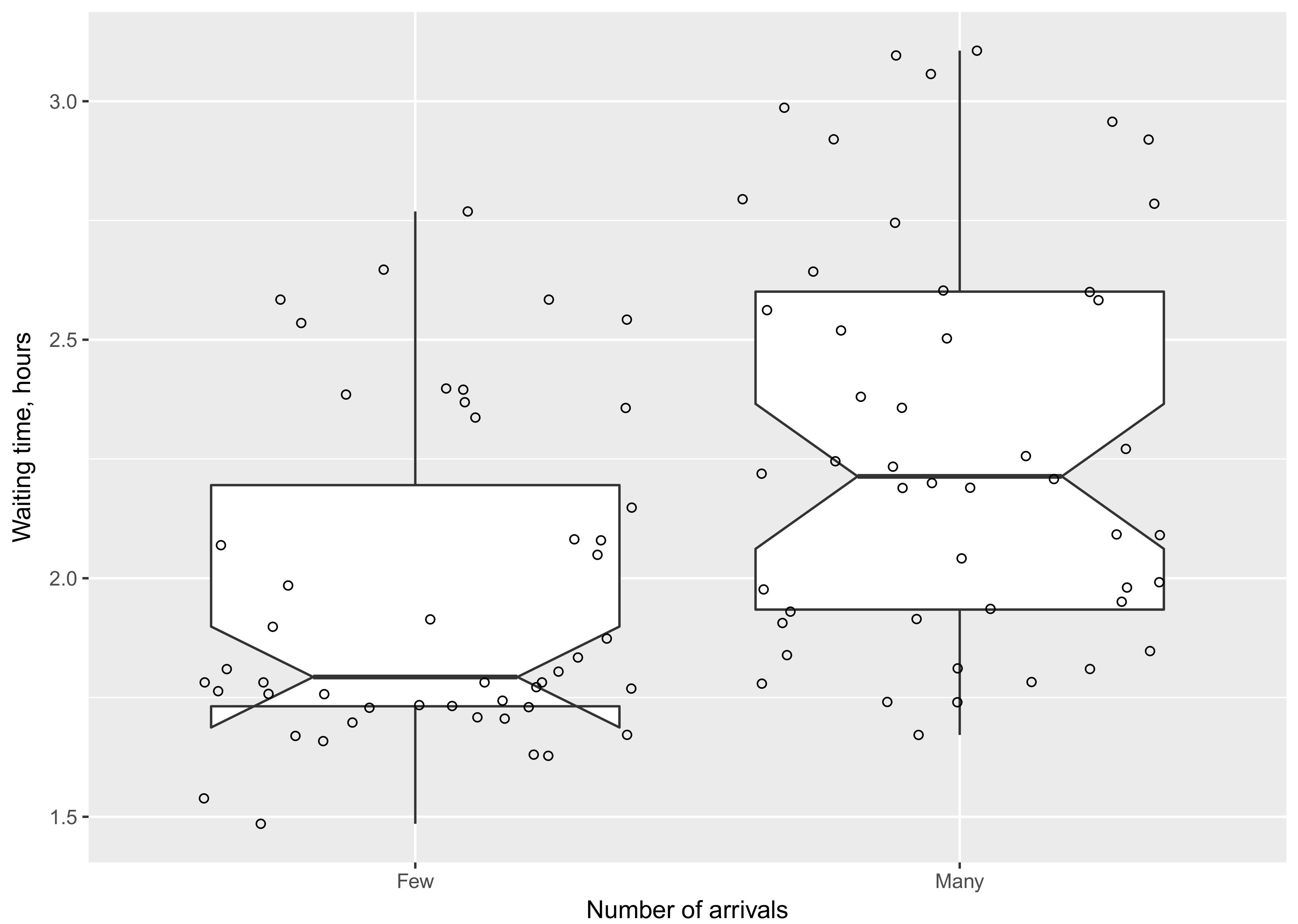

Supplement: Supplementary file 5 — Boxplot, illustrating the median and inter quartile range (IQR) of the waiting time on the 20th October and 5th September 2013. The median estimated waiting time was 1 h and 48 min (IQR = 27 min) on the 20th October and 2 h and 13 min (IQR = 40 min) on the 5th September. The notch indicates the estimated 95% confidence interval for the median. The individual observations are jittered. See Additional file 4 for a table of arrivals, departures and expected waiting time for each 30 min time interval. (TIF 425 kb) [file 12874_2019_710_MOESM5_ESM.tif]
