# Supplementary material for: Crowding in the emergency department in the absence of boarding – a transition regression model to predict departures and waiting time
Source: BMC Med Res Methodol. 2019 Mar 29;19:68. doi: 10.1186/s12874-019-0710-3 (PMC6440135; doi:10.1186/s12874-019-0710-3)
Supplement: Supplementary file 2 — Figure of diagnostic plot for the validation data (six month of 2014). See main text, Figs. 1 and 2, and Additional file 3 for comparison to 2013 data. See Additional file 3 for plots of arrivals, departures and queue length. (PDF 1495 kb) [file 12874_2019_710_MOESM2_ESM.pdf]

WEEKDAY

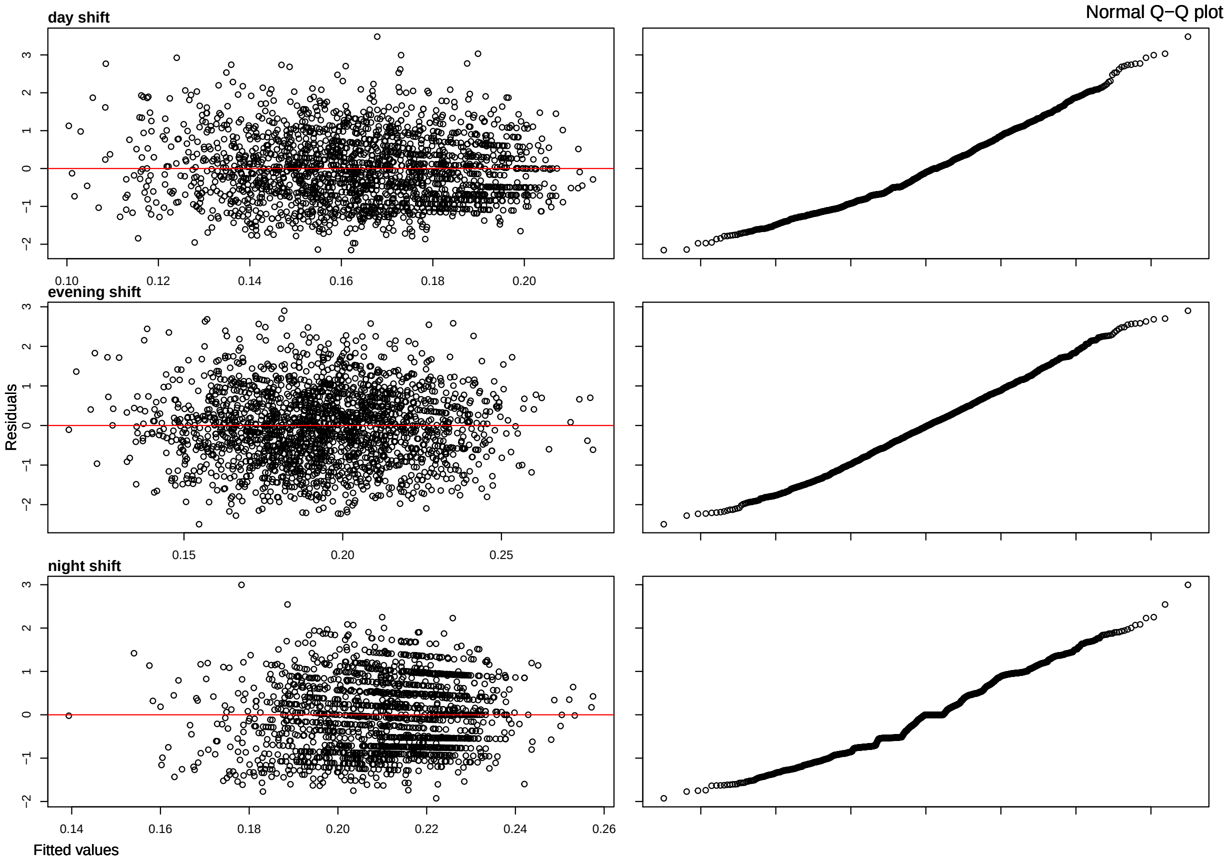

WEEKEND

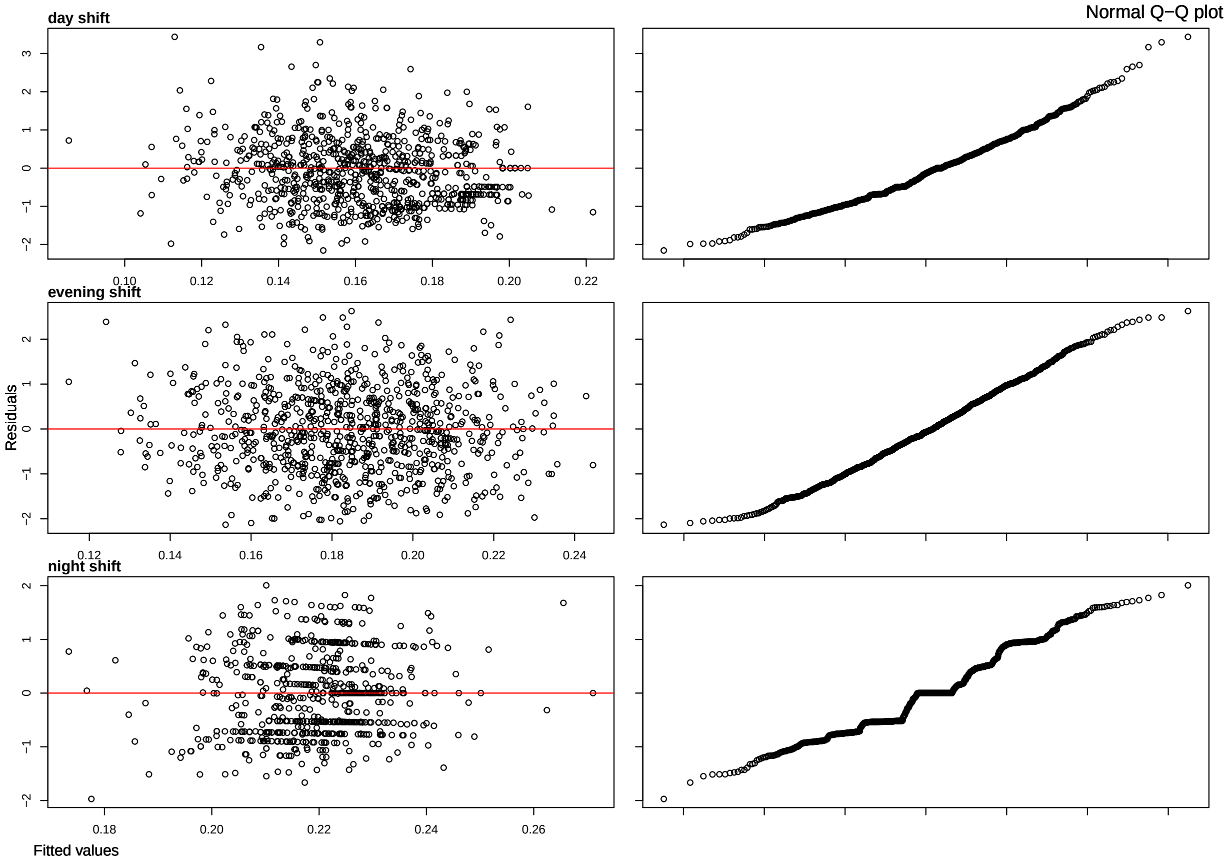

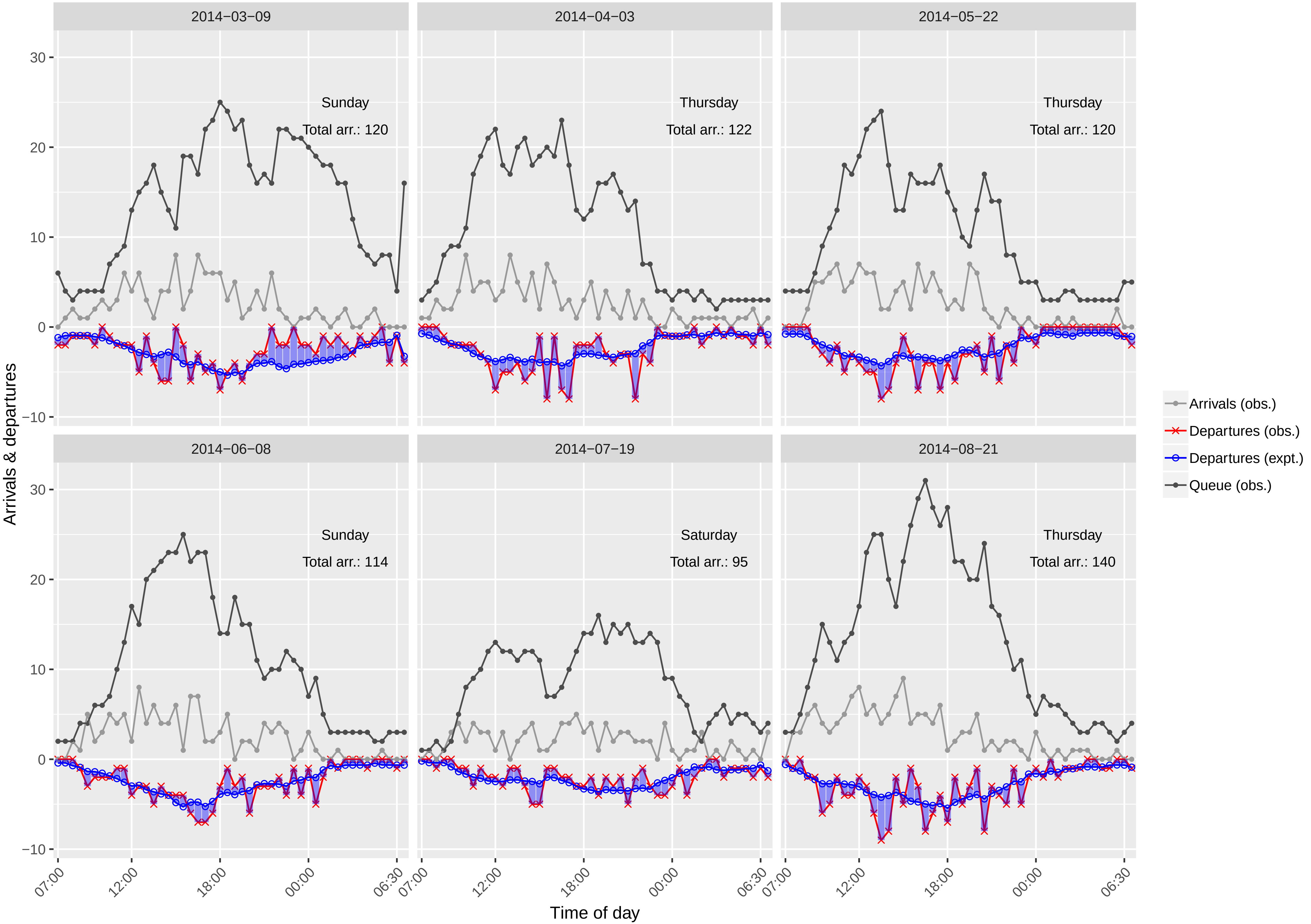
2014−03−09
2014−04−03
2014−05−22
2014−06−08
2014−07−19
2014−08−21
Sunday
Total arr.: 120
Thursday
Total arr.: 122
Thursday
Total arr.: 120
Sunday
Total arr.: 114
Saturday
Total arr.: 95
Thursday
Total arr.: 140
Arrivals & departures
Time of day
Arrivals (obs.)
Departures (obs.)
Departures (expt.)
Queue (obs.)
30
20
10
0
−10
07:00
12:00
18:00
00:00
06:30

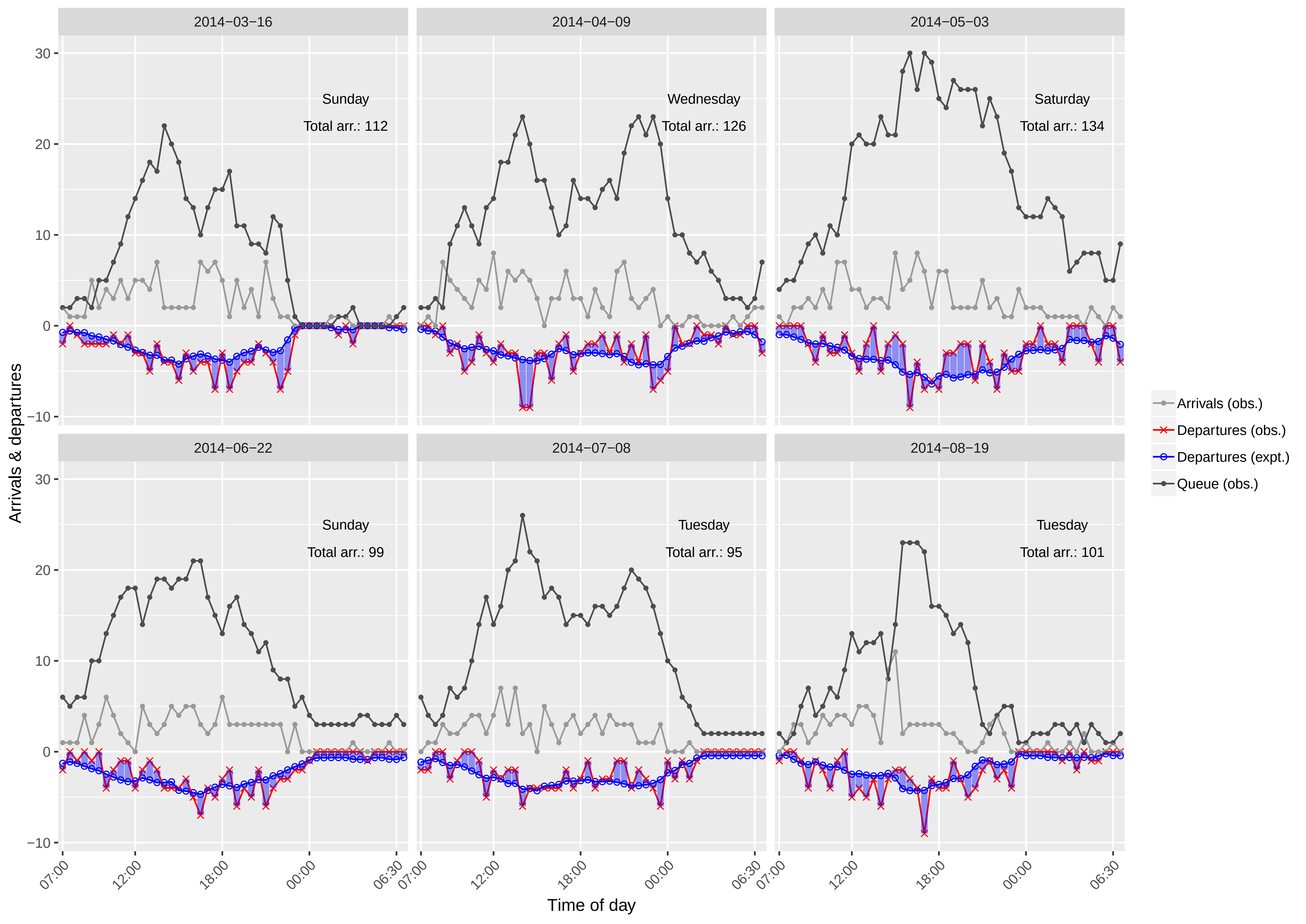

2014−03−16
2014−04−09
2014−05−03
2014−06−22
2014−07−08
2014−08−19
Sunday
Total arr.: 112
Wednesday
Total arr.: 126
Saturday
Total arr.: 134
Sunday
Total arr.: 99
Tuesday
Total arr.: 95
Tuesday
Total arr.: 101
Arrivals (obs.)
Departures (obs.)
Departures (expt.)
Queue (obs.)
Arrivals & departures
Time of day
30
20
10
0
−10
07:00
12:00
18:00
00:00
06:30

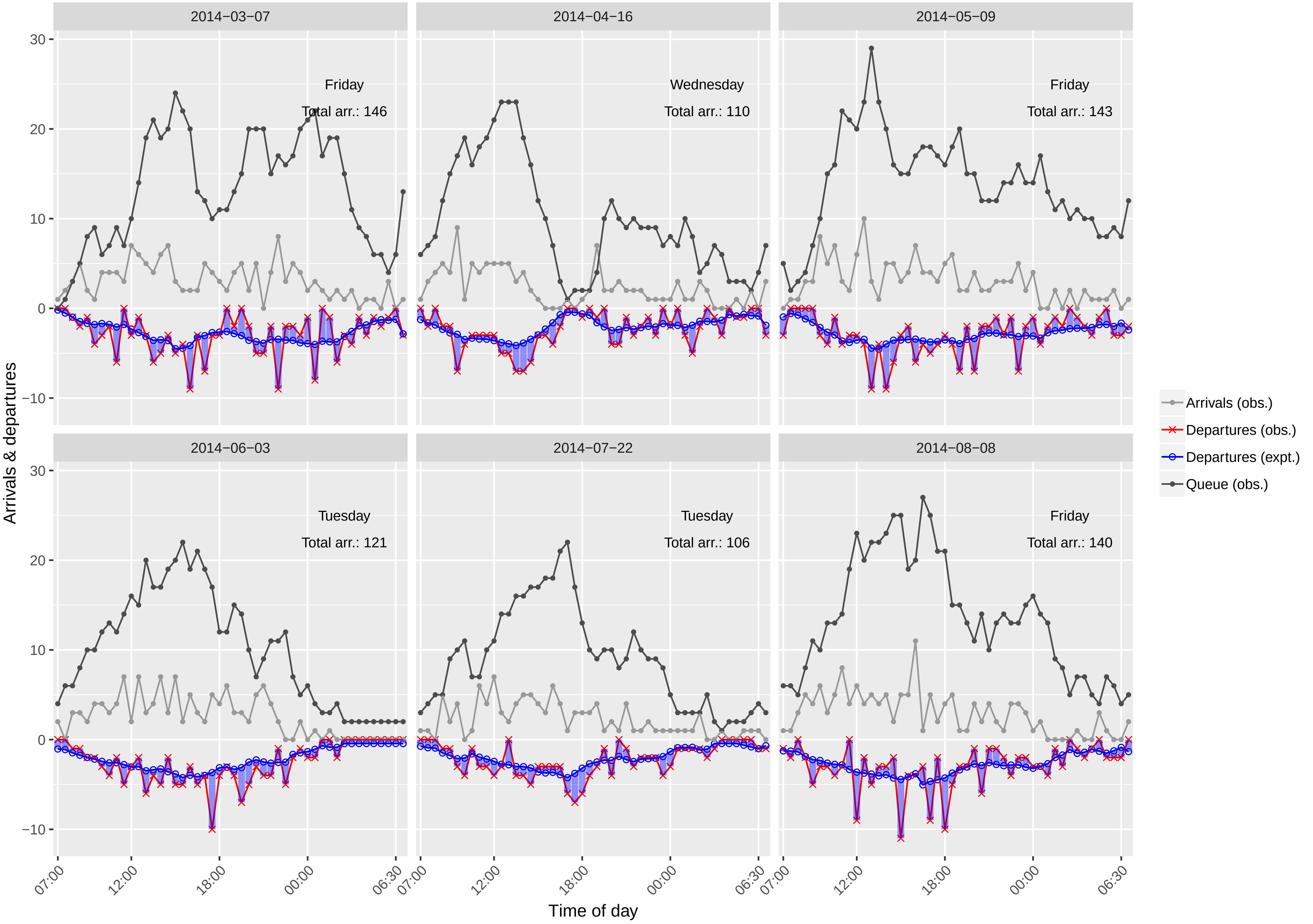

2014−03−07
2014−04−16
2014−05−09
2014−06−03
2014−07−22
2014−08−08
Friday
Total arr.: 146
Wednesday
Total arr.: 110
Friday
Total arr.: 143
Tuesday
Total arr.: 121
Tuesday
Total arr.: 106
Friday
Total arr.: 140
30
20
10
0
−10
07:00
12:00
18:00
00:00
06:30
Arrivals & departures
Time of day
Arrivals (obs.)
Departures (obs.)
Departures (expt.)
Queue (obs.)

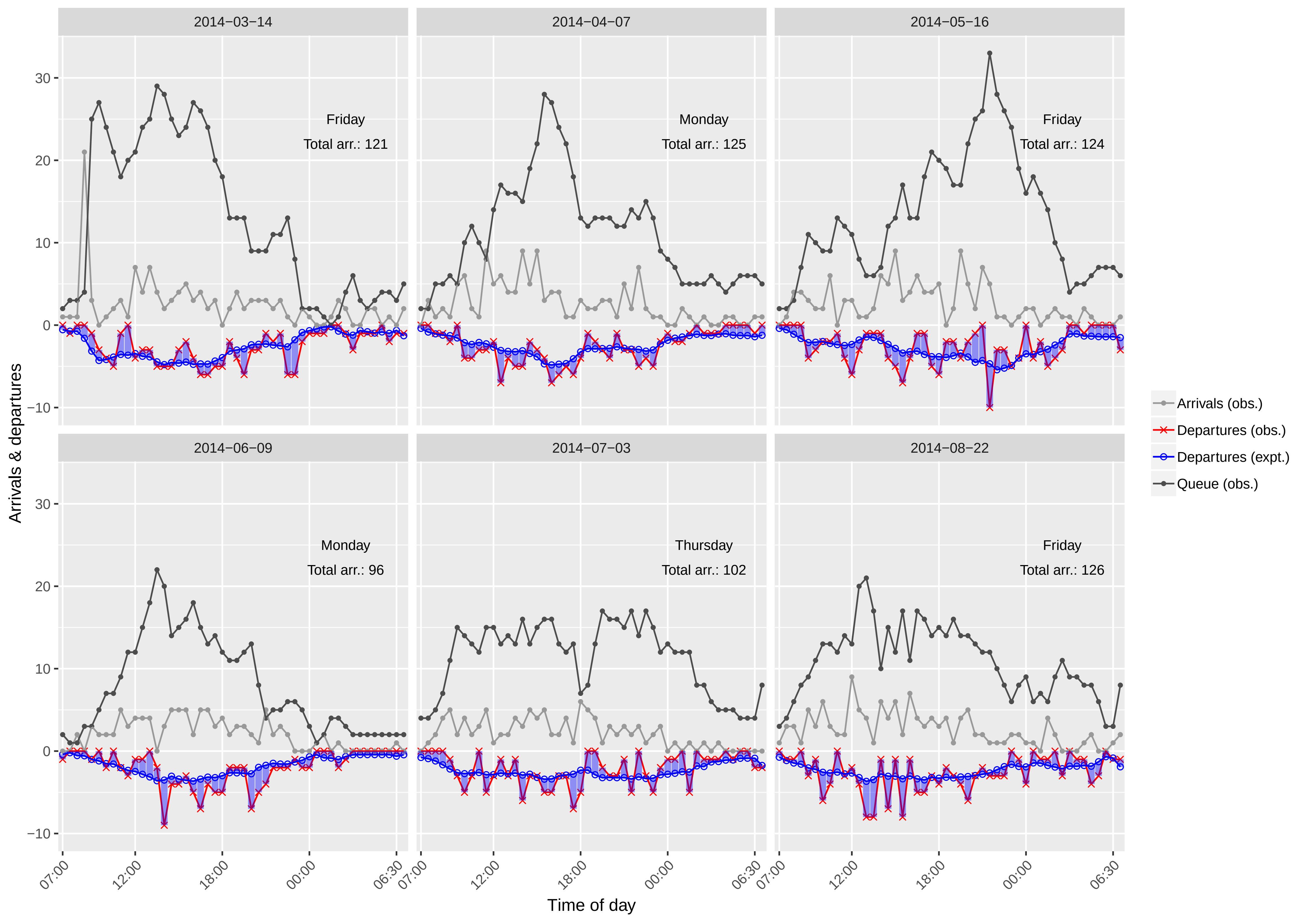
2014−03−14
2014−04−07
2014−05−16
2014−06−09
2014−07−03
2014−08−22
Friday
Total arr.: 121
Monday
Total arr.: 125
Friday
Total arr.: 124
Monday
Total arr.: 96
Thursday
Total arr.: 102
Friday
Total arr.: 126
Arrivals & departures
Time of day
07:00
12:00
18:00
00:00
06:30
30
20
10
0
−10
Arrivals (obs.)
Departures (obs.)
Departures (expt.)
Queue (obs.)

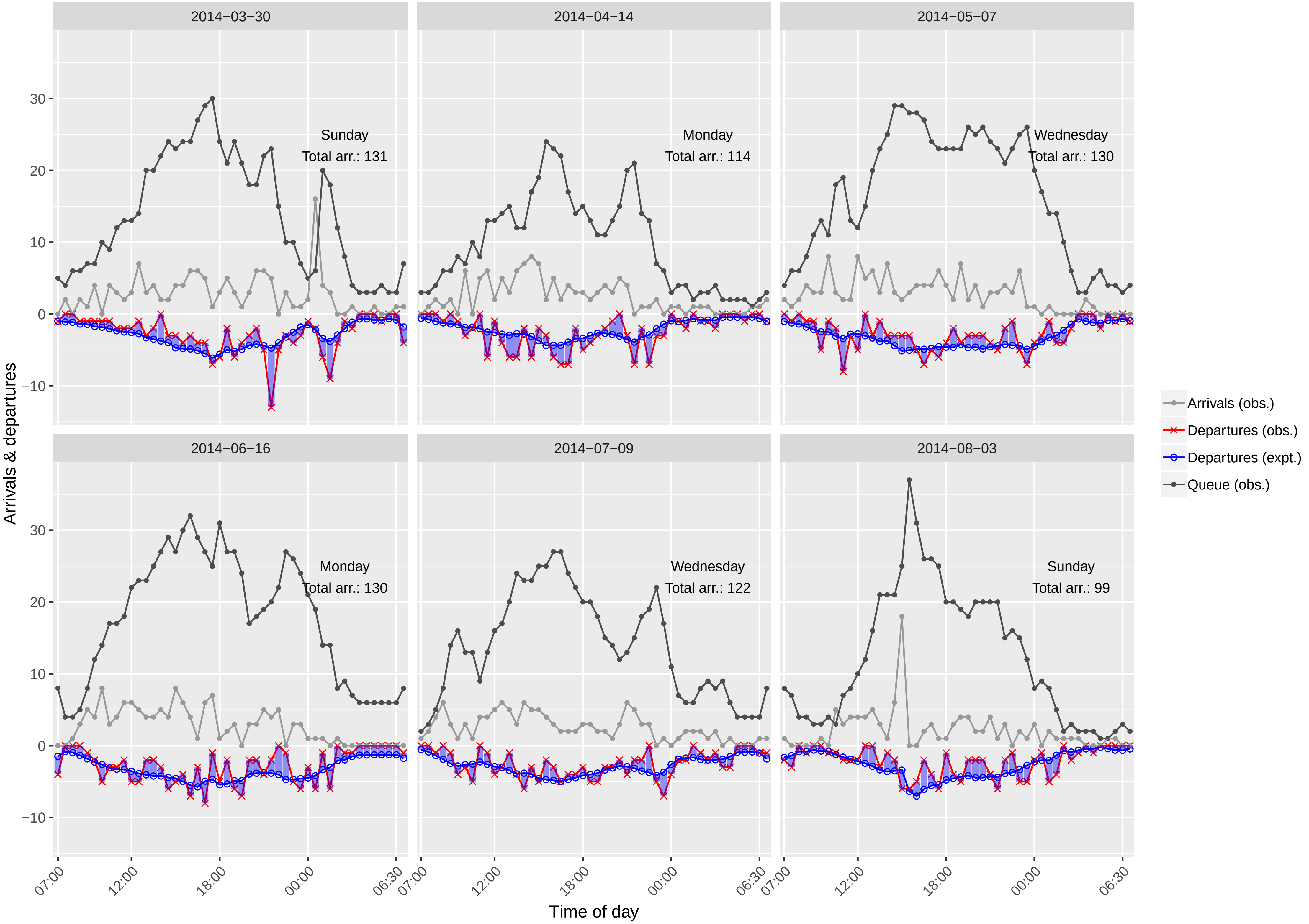

2014−03−30
2014−04−14
2014−05−07
2014−06−16
2014−07−09
2014−08−03
Sunday
Total arr.: 131
Monday
Total arr.: 114
Wednesday
Total arr.: 130
Monday
Total arr.: 130
Wednesday
Total arr.: 122
Sunday
Total arr.: 99
Arrivals & departures
30
20
10
0
−10
Time of day
07:00
12:00
18:00
00:00
06:30
Arrivals (obs.)
Departures (obs.)
Departures (expt.)
Queue (obs.)

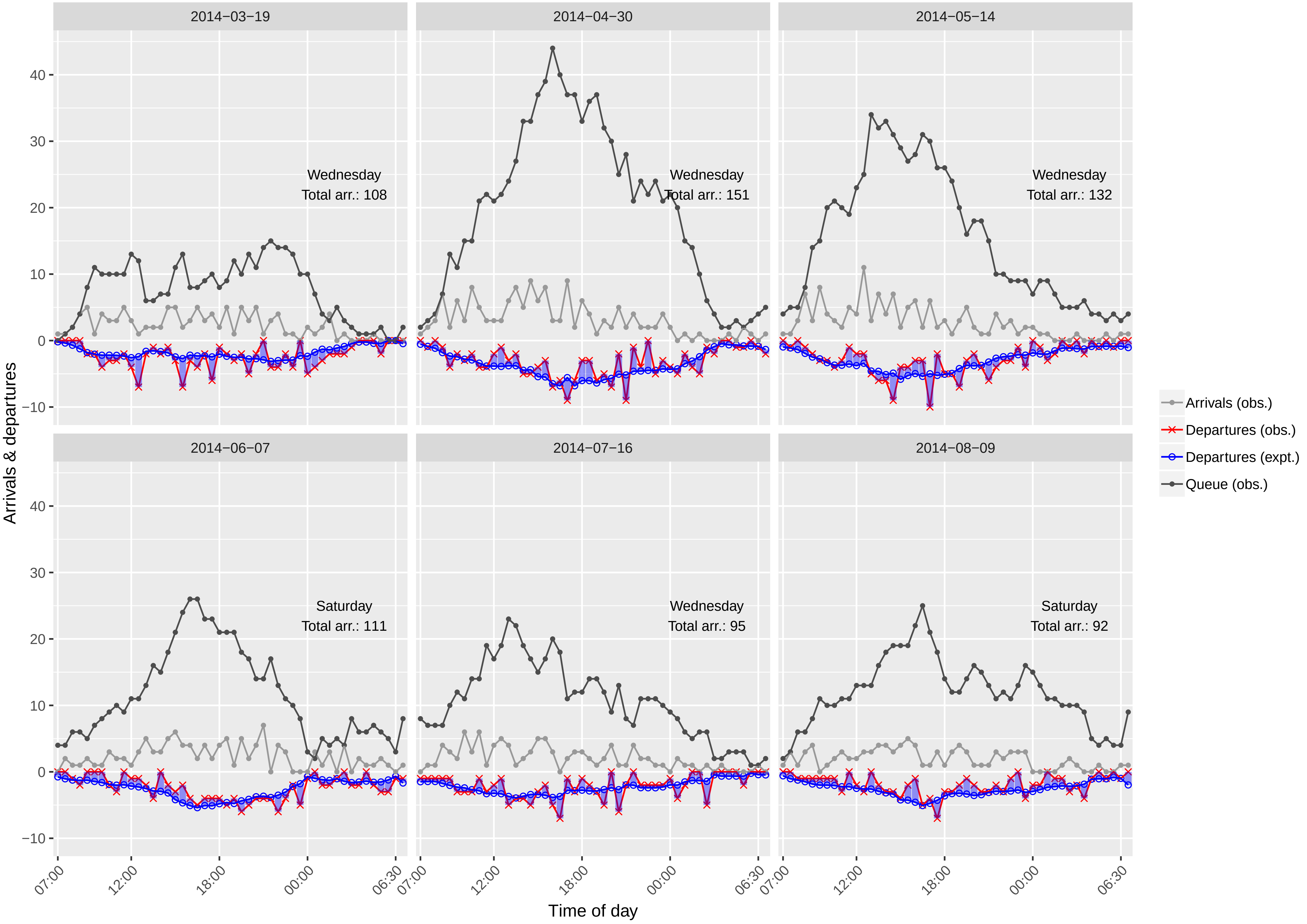
2014−03−19
2014−04−30
2014−05−14
2014−06−07
2014−07−16
2014−08−09
Wednesday
Total arr.: 108
Wednesday
Total arr.: 151
Wednesday
Total arr.: 132
Saturday
Total arr.: 111
Wednesday
Total arr.: 95
Saturday
Total arr.: 92
Arrivals & departures
Time of day
40
30
20
10
0
−10
07:00
12:00
18:00
00:00
06:30
Arrivals (obs.)
Departures (obs.)
Departures (expt.)
Queue (obs.)

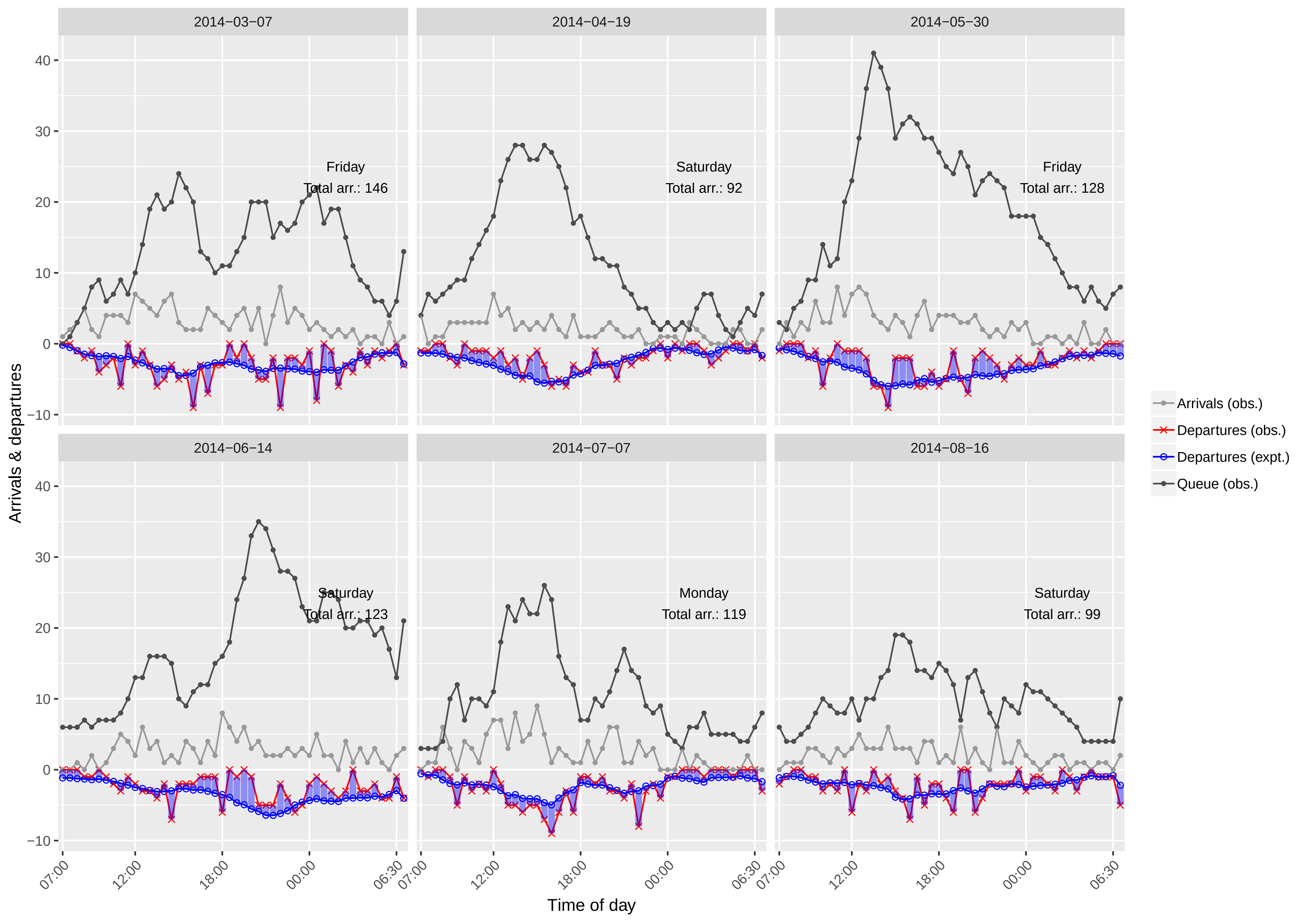
2014−03−07
2014−04−19
2014−05−30
2014−06−14
2014−07−07
2014−08−16
Friday
Total arr.: 146
Saturday
Total arr.: 92
Friday
Total arr.: 128
Saturday
Total arr.: 123
Monday
Total arr.: 119
Saturday
Total arr.: 99
Arrivals & departures
Time of day
07:00
12:00
18:00
00:00
06:30
Arrivals (obs.)
Departures (obs.)
Departures (expt.)
Queue (obs.)

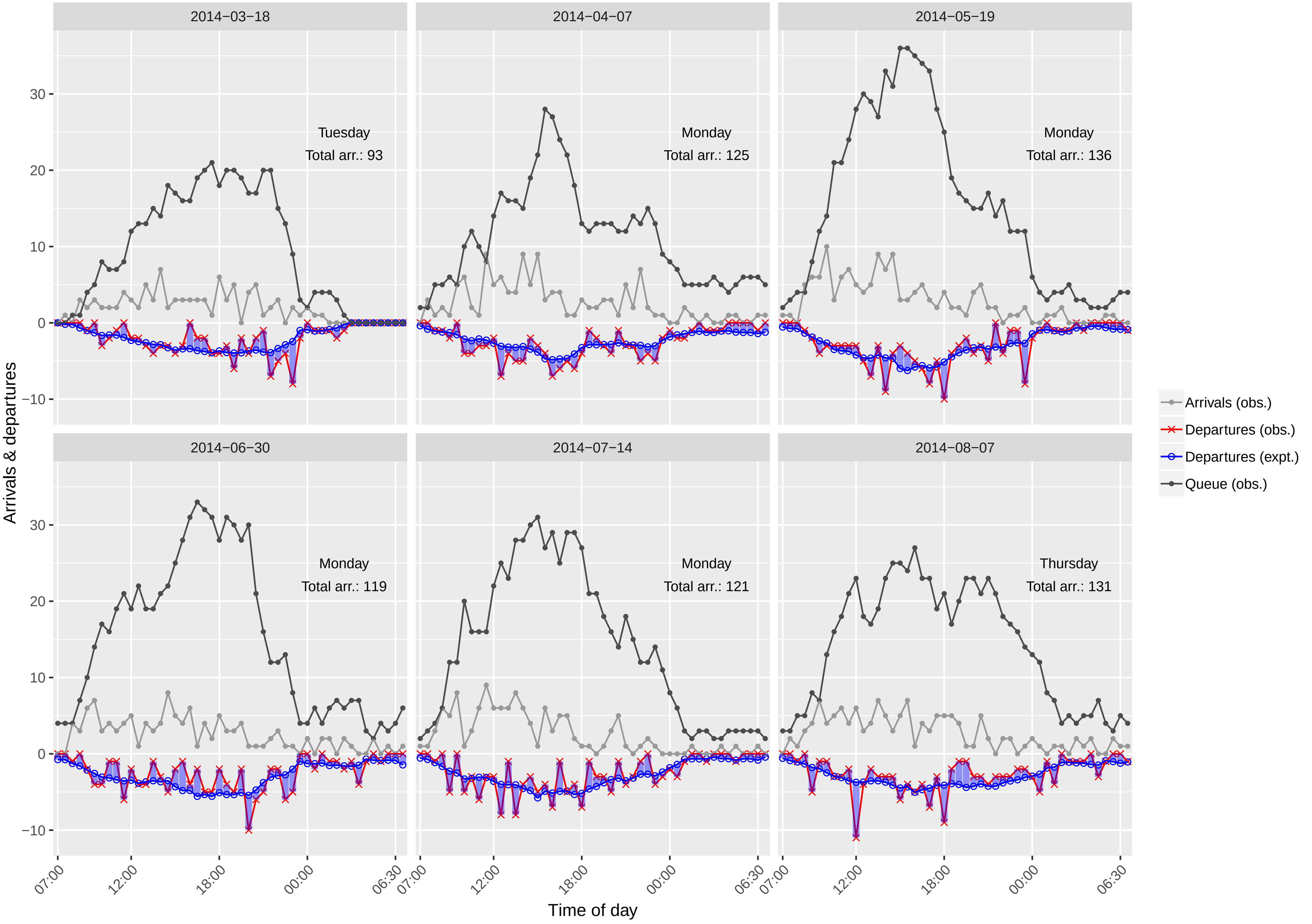
2014−03−18
2014−04−07
2014−05−19
2014−06−30
2014−07−14
2014−08−07
Tuesday
Total arr.: 93
Monday
Total arr.: 125
Monday
Total arr.: 136
Monday
Total arr.: 119
Monday
Total arr.: 121
Thursday
Total arr.: 131
Arrivals & departures
Time of day
07:00
12:00
18:00
00:00
06:30
30
20
10
0
−10
Arrivals (obs.)
Departures (obs.)
Departures (expt.)
Queue (obs.)
