# Supplementary material for: Crowding in the emergency department in the absence of boarding – a transition regression model to predict departures and waiting time
Source: BMC Med Res Methodol. 2019 Mar 29;19:68. doi: 10.1186/s12874-019-0710-3 (PMC6440135; doi:10.1186/s12874-019-0710-3)
Supplement: Supplementary file 4 — Table of the expected waiting time in a scenario with few and many arrivals. Two examples of the number of arrivals and departures, the resulting queue length and probability of departure, and the waiting time estimate based on this data. The examples are chosen to show the contrast of a day with few arrivals and a day with many arrivals (the graphs of predicted arrivals etc. can be seen in Additional file 3). It exemplifies how arrivals can drive the waiting time e.g. in the time interval beginning at 15:00: The queue length were the same and twice as many (2 and 4, respectively) left the ED on the 5th September. But while there were four patients that arrived on the 5th eight arrived on the 20th. This resulted in a waiting time estimate of 2 h and 39 min and 1 h and 59 min, respectively. It is also clear that arrivals are not the only factor that influence waiting time. The change in queue length is seen to be important exemplified in the time intervals beginning at 7:30 and 8:00 both on the 5th September. Here nothing but the queue length changed (rises from 1 to 3 patients, respectively) and the waiting time increasing with 5 min. See Additional file 5 for a boxplot of the waiting time. *Waiting time estimated in hours as 0.5 * (1 – p) / p, where p is the probability of a departure. (PDF 41 kb) [file 12874_2019_710_MOESM4_ESM.pdf]

	80 arrivals, 20th October 2013				147 arrivals, 5th September 2013				Estimated waiting time*	
**Time of day**	Arrivals	Departures	Queue length	Probability of departure	Arrivals	Departures	Queue length	Probability of departure	20th October	5th September
**07:00-07:29**	0	0	0	0,207	1	0	0	0,197	1,914	2,042
**07:30-07:59**	1	0	0	0,195	2	0	1	0,180	2,069	2,271
**08:00-08:29**	1	0	1	0,189	2	0	3	0,175	2,148	2,357
**08:30-08:59**	0	0	2	0,194	6	1	5	0,139	2,082	3,106
**09:00-09:29**	5	0	2	0,153	2	4	10	0,159	2,769	2,643
**09:30-09:59**	2	0	7	0,162	4	6	8	0,162	2,584	2,583
**10:00-10:29**	2	2	9	0,165	3	3	6	0,182	2,535	2,245
**10:30-10:59**	3	3	9	0,162	3	1	6	0,183	2,584	2,234
**11:00-11:29**	2	5	9	0,175	4	1	8	0,163	2,357	2,562
**11:30-11:59**	1	4	6	0,201	5	6	11	0,143	1,985	2,986
**12:00-12:29**	5	2	3	0,173	4	5	10	0,161	2,385	2,600
**12:30-12:59**	3	3	6	0,174	8	4	9	0,139	2,369	3,096
**13:00-13:29**	1	3	6	0,194	6	3	13	0,145	2,080	2,957
**13:30-13:59**	5	3	4	0,164	4	5	16	0,154	2,542	2,745
**14:00-14:29**	3	5	6	0,173	4	8	15	0,161	2,395	2,603
**14:30-14:59**	4	0	4	0,176	3	6	11	0,186	2,337	2,190
**15:00-15:29**	8	4	8	0,159	4	2	8	0,201	2,647	1,992
**15:30-15:59**	1	4	12	0,214	3	3	10	0,193	1,834	2,092
**16:00-16:29**	2	6	9	0,224	8	4	10	0,146	1,729	2,920
**16:30-16:59**	4	3	5	0,222	7	5	14	0,141	1,757	3,057
**17:00-17:29**	0	1	6	0,252	2	3	16	0,186	1,485	2,189
**17:30-17:59**	2	2	5	0,230	7	3	15	0,152	1,672	2,795
**18:00-18:29**	2	1	5	0,220	5	3	19	0,152	1,772	2,785
**18:30-18:59**	0	2	6	0,235	3	5	21	0,167	1,630	2,503
**19:00-19:29**	1	1	4	0,235	7	6	19	0,146	1,628	2,920
**19:30-19:59**	7	5	4	0,173	2	3	20	0,185	2,398	2,200
**20:00-20:29**	2	2	6	0,216	5	6	19	0,166	1,810	2,519
**20:30-20:59**	2	2	6	0,224	1	6	18	0,202	1,734	1,977
**21:00-21:29**	0	1	6	0,245	5	7	13	0,181	1,539	2,256
**21:30-21:59**	2	2	5	0,221	4	3	11	0,193	1,758	2,090
**22:00-22:29**	3	1	5	0,208	2	1	12	0,202	1,898	1,981
**22:30-22:59**	1	3	7	0,220	4	4	13	0,174	1,769	2,380
**23:00-23:29**	0	1	5	0,226	3	7	13	0,184	1,708	2,219
**23:30-23:59**	3	4	4	0,196	3	2	9	0,204	2,049	1,951
**00:00-00:29**	0	1	3	0,230	1	2	10	0,216	1,670	1,811
**00:30-00:59**	1	1	2	0,221	1	1	9	0,216	1,763	1,810
**01:00-01:29**	0	2	2	0,228	1	1	9	0,205	1,698	1,936
**01:30-01:59**	0	0	0	0,232	3	4	9	0,185	1,659	2,208
**02:00-02:29**	0	0	0	0,227	0	1	8	0,219	1,706	1,782
**02:30-02:59**	0	0	0	0,224	0	1	7	0,223	1,732	1,741
**03:00-03:29**	1	0	0	0,211	0	3	6	0,223	1,874	1,740
**03:30-03:59**	0	0	1	0,217	0	0	3	0,230	1,804	1,672
**04:00-04:29**	0	0	1	0,219	1	0	3	0,214	1,782	1,839
**04:30-04:59**	0	0	1	0,219	1	1	4	0,207	1,782	1,915
**05:00-05:29**	0	0	1	0,219	1	1	4	0,206	1,782	1,930
**05:30-05:59**	0	1	1	0,219	1	1	4	0,208	1,782	1,906
**06:00-06:29**	0	0	0	0,224	0	1	4	0,219	1,730	1,779
**06:30-06:59**	0	0	0	0,223	1	0	3	0,213	1,743	1,847
